# Avian Toll-like receptor allelic diversity far exceeds human polymorphism: an insight from domestic chicken breeds

**DOI:** 10.1038/s41598-018-36226-1

**Published:** 2018-12-14

**Authors:** Zuzana Świderská, Adéla Šmídová, Lucie Buchtová, Anna Bryjová, Anežka Fabiánová, Pavel Munclinger, Michal Vinkler

**Affiliations:** 1 Charles University, Faculty of Science, Department of Zoology, Viničná 7, Prague, 12843 Czech Republic; 2 Charles University, Faculty of Science, Department of Cell Biology, Viničná 7, Prague, 12843 Czech Republic; 3 The Czech Academy of Sciences, Institute of Vertebrate Biology, v.v.i., Květná 8, Brno, 60365 Czech Republic

## Abstract

Immune genes show remarkable levels of adaptive variation shaped by pathogen-mediated selection. Compared to humans, however, population polymorphism in animals has been understudied. To provide an insight into immunogenetic diversity in birds, we sequenced complete protein-coding regions of all Toll-like receptor (*TLR*) genes with direct orthology between mammals and birds (*TLR3*, *TLR4*, *TLR5* and *TLR7*) in 110 domestic chickens from 25 breeds and compared their variability with a corresponding human dataset. Chicken *TLRs* (*chTLRs*) exhibit on average nine-times higher nucleotide diversity than human *TLRs* (*hTLRs*). Increased potentially functional non-synonymous variability is found in *chTLR* ligand-binding ectodomains. While we identified seven sites in *chTLRs* under positive selection and found evidence for convergence between alleles, no selection or convergence was detected in *hTLRs*. Up to six-times more alleles were identified in fowl (70 *chTLR4* alleles vs. 11 *hTLR4* alleles). In *chTLRs*, high numbers of alleles are shared between the breeds and the allelic frequencies are more equal than in *hTLRs*. These differences may have an important impact on infectious disease resistance and host-parasite co-evolution. Though adaptation through high genetic variation is typical for acquired immunity (e.g. *MHC*), our results show striking levels of intraspecific polymorphism also in poultry innate immune receptors.

## Introduction

Domestic chickens and humans have a lot in common. Given their joint history, the domestic chicken is now an abundant and widespread species around the world. Both humans and chickens now serve as basic research model species representing genome references for birds and mammals. Yet domestic chicken breeds remain rarely studied from the perspective of evolutionary immunology. This is despite Darwin himself pointed out that variation in domestic fowl populations provides an excellent system for investigating evolution through natural and artificial selection^1^. Selection is particularly strong at shaping host-pathogen interactions, where different genes can be selected for genetic diversity. In the light of these facts, it is surprising how little attention has been paid to immunologically-relevant genetic variation in the highly diversified yet phenotypically standardised breeds of domestic fowl^2–5^.

Unlike the many laboratory and commercial lines more typically studied^6,7^, many domestic chicken breeds exhibit high genetic and phenotypic variation^8–10^. Several hundreds of breeds are now recognised worldwide, many of which are only locally distributed, having been maintained as stable phenotypic forms for centuries^11^. These traditional fowl breeds were originally domesticated from free-living red junglefowl subspecies, with possible admixture of other *Gallus* species, on multiple occasions in different regions of Asia^12^. This, together with distinct trade-driven migration routes and selection for different human needs in different environments, may have diversified the breeds phenotypically as regards pathogen resistance.

Previous studies suggest that the chicken genome is approximately two-times richer in exon polymorphism than the human genome^13,14^. From an evolutionary perspective, variation in innate immune receptor genes, which form a direct molecular interface between pathogens and their hosts, is particularly appealing since major evolutionary adaptations among polymorphic variants can be predicted^15^. Toll-like receptors (TLRs) act as innate immunity sensors responsible for detection of invading pathogen ligands during early phases of an infection^16^. TLRs are type I transmembrane proteins present either on the cell surface or in the intracellular compartments. They typically consist of a pathogen-recognition horseshoe-shaped ectodomain, a short segment spanning the membrane and an intracellular toll/interleukin-1 receptor (TIR) signalling domain^17^.

TLRs are encoded by a multigene family which is only partially conserved across vertebrates, e.g. people and chickens have similar numbers of TLR genes^18^, but only four functionally distinct TLRs show direct orthology between both species^19^: endosomal viral-dsRNA-sensing TLR3^20,21^; TLR4 detecting bacterial lipopolysaccharide (LPS) and various other pathogen-derived and host-derived compounds on cell surfaces^22,23^; cell-surface-based bacterial-flagellin-sensing TLR5^24,25^; and endosomal viral-ssRNA-sensing TLR7^26^. The other TLRs may be duplicated (e.g. chicken TLR1 and TLR2^27^), pseudogenised (chicken TLR8^26^) or unique in either of the species (e.g. human TLR9^28^; or chicken TLR15^29^ and TLR21^30^). Although human TLR7 and TLR8 are closely related, they slightly differ in their natural ligand preferences^31–34^.

Although usually unable to avoid expression of TLR ligands, pathogens in many cases have succeeded in evolving structural modifications that impair recognition by TLRs^35^. Co-evolution with pathogens can then select for diversification in TLR alleles through specific adaptations to ligand variants^15^. Accordingly, most parts of the TLR molecule remain highly conservative due to purifying selection, while other parts, such as the ligand-binding regions, exhibit striking variability, both at the interspecific and intraspecific levels^36–40^. This variation could affect disease resistance^41,42^.

In this study, we compare genetic variability and evolutionary patterns in *TLR3*, *TLR4*, *TLR5* and *TLR7* in humans (the only other species with large-sample intraspecific TLR diversity data publicly available), represented by 25 world-wide populations, and domestic chickens, represented by 25 traditional breeds. Information on sequence variation in these receptors is used to show differences in levels of potentially functional variation and the number of sites under positive selection between humans and domestic chickens. Furthermore, we also compare data on allele frequencies and allele sharing. Besides this, we examined the patterns of *TLR* variation with respect to a neutral mitochondrial marker and linked population structure in chicken *TLRs* (*chTLR*) to neutral population structure based on 19 microsatellites (allowing us comparison with previously published evidence for chicken breeds). In doing so, this study provides a pioneering insight into understanding the remarkable levels of variation in non-human immunogenetics when investigated outside traditional commercial and inbred models.

## Results

### Comparison of genetic variation in human and chicken *TLRs*

**Figure 1:**
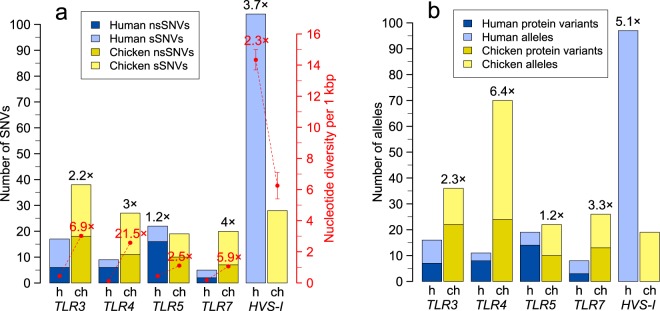
Genetic variability in human and chicken Toll-like receptors (*TLRs*) and hypervariable segment I (*HVS-I*) of the mitochondrial DNA. (**a**) Comparison of numbers of single nucleotide variants (SNVs; left axis) and nucleotide diversity (right axis; red) found in human (h; blue) and chicken (ch; yellow) *TLRs* and *HVS-I*. Synonymous variants are shown in light colours and non-synonymous variants in dark colours. (**b**) Comparison of numbers of alleles and protein variants of *TLRs* found in human (h; blue) and chicken (ch; yellow) populations and number of alleles found in *HVS-I*. Total numbers of TLR protein variants are highlighted in dark colours, while numbers of alleles are represented by the total height of each bar (combination of light and dark colours). Numbers above the bars and points indicate the fold differences between values for humans and chickens. Error bars denote standard deviations (in *TLR* genes the bars are too short to be visible).

**Figure 2:**
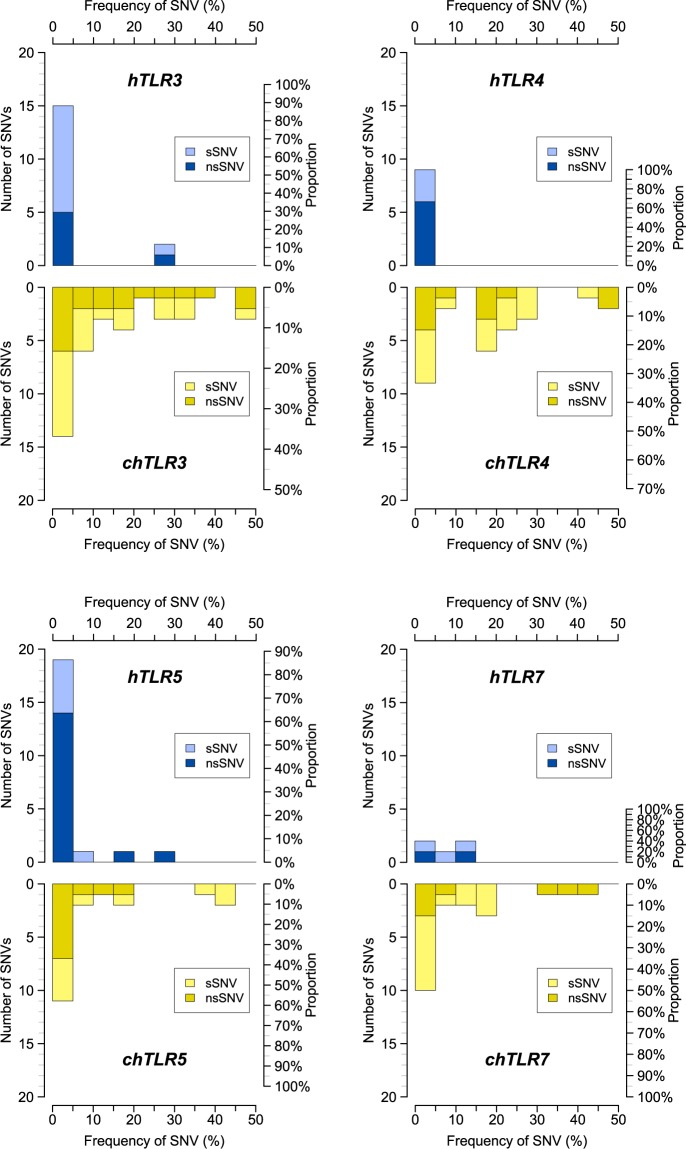
Comparison of minor variant frequencies in human and chicken *TLR* single nucleotide variants (SNVs). Human *TLRs* (*hTLRs*) are highlighted in blue and chicken *TLRs* (*chTLRs*) in yellow, while allele frequencies of equivalent TLRs are shown specularly. Synonymous variants are shown in light colours and non-synonymous variants in dark colours. The left axis shows number of SNVs, while the right axis indicates the proportion of SNVs on a relative scale.

We sequenced complete protein-coding DNA sequences (CDSs) for all *TLRs* with direct orthologues between mammals and birds (*TLR3*, *TLR4*, *TLR5* and *TLR7*) in 110 chickens (25 breeds) to compare variability with a corresponding set of 110 randomly-selected humans sampled from 25 populations around the world as part of the 1000 Genomes Project^43^. For a complete description of SNVs detected in chicken *TLRs* (*chTLR*), including their frequencies, see Supplementary Table S1. In most *chTLRs* (*TLR3*, *TLR4* and *TLR7*) we observed between 2.2× and 4× more single nucleotide variants (SNVs) than in human *TLRs* (*hTLRs*), with 17 SNVs in *hTLR3* vs 38 SNVs in *chTLR3*; 9 SNVs in *hTLR4* vs 27 SNVs in *chTLR4*; and 5 SNVs in *hTLR7* vs 20 SNVs in *chTLR7* (Fig. 1a, Table 1). Only in *TLR5* did we detect slightly more SNVs in *hTLR5* (22 SNVs) than in c*hTLR5* (19 SNVs). Interestingly, 86% of the *hTLR5* SNVs were very rare variants with frequencies below 5%, while only 58% of the SNVs were below 5% frequency in *chTLR5* (Fig. 2). This is reflected in the nucleotide diversity (π), which was 2.5× higher in *chTLR5* than *hTLR5* (Fig. 1a, Table 1). While the frequency of SNVs in all *hTLR* genes was highly skewed, those found in *chTLRs* had a more equal variant representation and more SNVs of medium frequencies. This was also true for non-synonymous single nucleotide variants (nsSNVs) potentially affecting TLR structure and function (Fig. 2). The higher number of SNVs and more equal variant frequency meant that *chTLRs* exhibited nucleotide diversity up to 21.5× higher than *hTLRs* (Table 1). Unlike *hTLRs*, we found no sequences with internal STOP codons in *chTLRs*. In contrast to *TLRs*, part of the mitochondrial control region previously used as a neutral marker of variability in humans (hypervariable segment I; *HVS-I*) harboured 3.7× more SNVs in humans than chickens (Fig. 1a). Our results do not appear to be affected by the particular selection of the 110 individuals in the chicken or human populations. In chickens, we tested the effect of variation in the numbers of individuals across the breeds on those breeds where we have at least 6 animals per breed represented in the data set (six breeds: Araucana AR, Brahma BH, Czech Golden Pencilled CZ, La Flèche LF, Rosecomb Bantam RO, and Sebright SE). The results show that the average chicken nucleotide diversity does not importantly change with the number of individuals used per breed (2, 4 or 6): *chTLR3* π_110_ = 0.00302, π_(2 ind × 6 br)_ = 0.00300, π_(4 ind × 6 br)_ = 0.00296, π_(6 ind × 6 br)_ = 0.00301; *chTLR4* π_110_ = 0.00258, π_(2 ind × 6 br)_ = 0.00241, π_(4 ind × 6 br)_ = 0.00250, π_(6 ind × 6 br)_ = 0.00244; *chTLR5* π_110_ = 0.00111, π_(2 ind × 6 br)_ =0.00122, π_(4 ind × 6 br)_ = 0.00114, π_(6 ind × 6 br)_ = 0.00117; *chTLR7* π_110_ = 0.00106, π_(2 ind × 6 br)_ = 0.00099, π_(4 ind × 6 br)_ =0.00100, π_(6 ind × 6 br)_ = 0.00096. Also increasing the human dataset to the full sample of 2504 people represented in the 1000 Genomes Project did not importantly alter our estimates of human *TLR* nucleotide diversity: *hTLR3* π_110_ = 0.00044 vs. π_2504_ = 0.00042, *hTLR4* π_110_ = 0.00012 vs. π_2504_ = 0.00016, *hTLR5* π_110_ = 0.00044 vs. π_2504_ = 0.00045, and *hTLR7* π_110_ = 0.00018 vs. π_2504_ = 0.00019.

**Table 1:** Diversity statistics of human and chicken *TLRs*.

Gene	Sp	L (bp)	SNVs	nsSNVs	k	π ± SD	A	PV	Hd ± SD
*TLR3*	h	2715	17	6	1.187	0.00044 ± 0.00002	16	7	0.759 ± 0.016
	ch	2691	38	18	8.120	0.00302 ± 0.00008	36	22	0.903 ± 0.011
*TLR4*	h	2520	9	6	0.310	0.00012 ± 0.00002	11	8	0.244 ± 0.038
	ch	2532	27	11	6.540	0.00258 ± 0.00005	70	24	0.947 ± 0.010
*TLR5*	h	2577	22	16	1.143	0.00044 ± 0.00003	19	14	0.709 ± 0.025
	ch	2586	19	10	2.866	0.00111 ± 0.00004	22	10	0.854 ± 0.013
*TLR7*	h	3150	5	2	0.566	0.00018 ± 0.00002	8	3	0.481 ± 0.037
	ch	3180	20	7	3.363	0.00106 ± 0.00005	26	13	0.849 ± 0.017

Sp: species (h: human, ch: chicken); L (bp): sequence length in base pairs; SNVs: number of single nucleotide variants; nsSNVs: number of non-synonymous single nucleotide variants; k: average number of nucleotide differences between two sequences; π: nucleotide diversity (average number of nucleotide differences per site between two sequences); SD: standard deviation; A: number of alleles; PV: number of protein variants; Hd: haplotype diversity.

### Location and physicochemical properties of the coding variants

**Figure 3:**
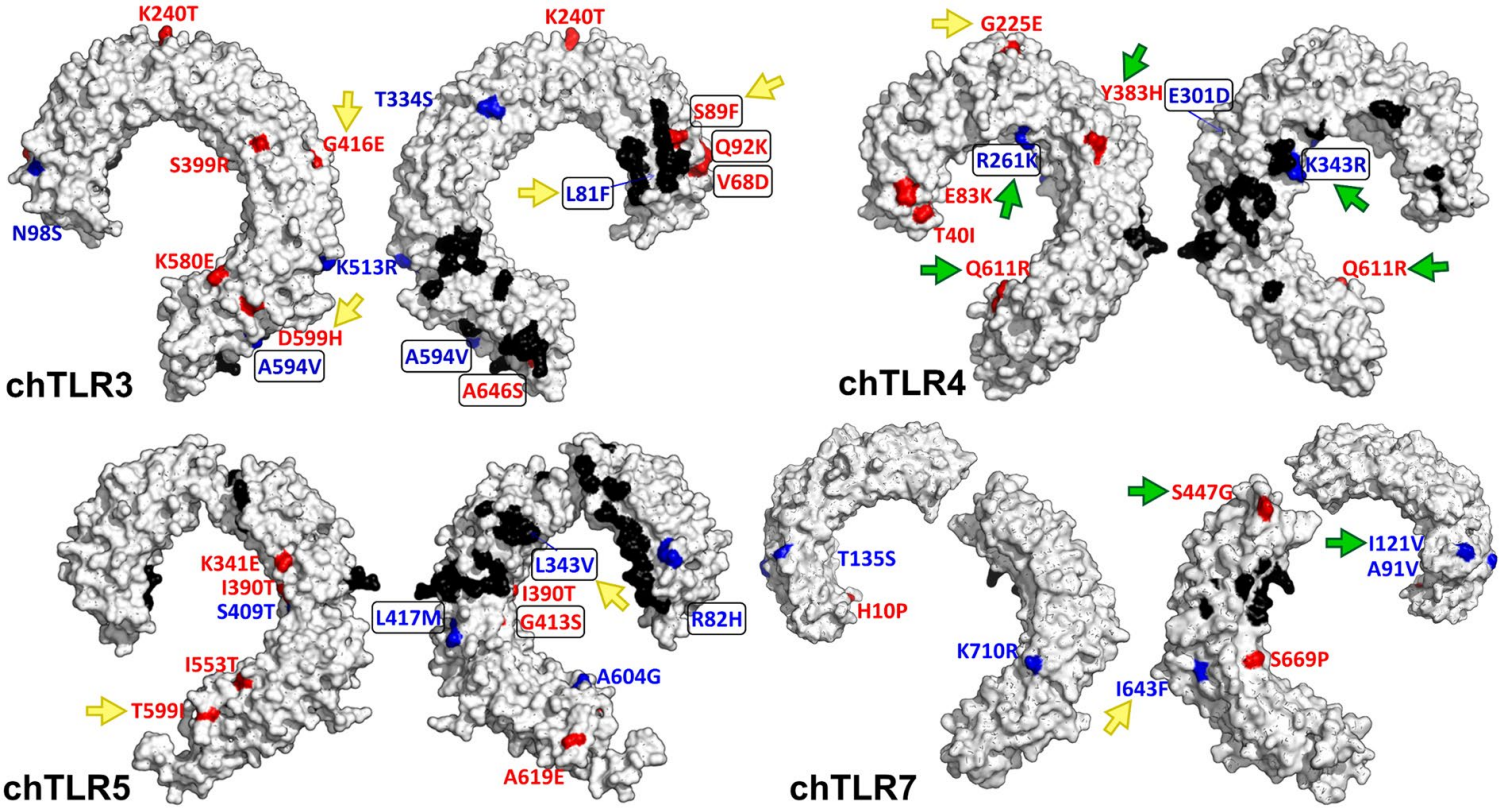
Projection of amino acid substitutions onto 3D models of chicken TLR ectodomains. Radical alternations are shown in red, conservative in blue and functionally important sites known from mammalian^68,90,91^ or fish^92^ studies are highlighted in black. Substitutions closer than 10 Å to functional sites are highlighted by a rectangle, non-conservative sites (PROVEAN) are indicated with yellow and positively selected sites (IFEL, FUBAR) with green arrows.

While nsSNVs were more evenly distributed across genes in *hTLRs*, almost all nsSNVs (with three exceptions, two in *chTLR3* and one in *chTLR7*) in *chTLRs* were located in the ligand-binding ectodomains (see Supplementary Fig. S1 and Supplementary Table S1). Based on 3D model measurements, thirteen chicken amino acid substitutions neighboured the predicted functional sites of TLRs with topological proximities of less than 10 Å (Fig. 3, Supplementary Table S1). Twelve nsSNVs found in *chTLR3*, six in *chTLR4*, six in *chTLR5* and two in *chTLR7* resulted in important differences in residue physicochemical properties, and hence may substantially influence the resultant ligand-binding features of the receptors (Fig. 3, Supplementary Table S1). Four amino acid substitutions in chTLR3, one in chTLR4, two in chTLR5 and one in chTLR7 were predicted by the PROVEAN analysis (score lower than the cut-off of −2.5) to affect the biological functions of the receptors (Fig. 3, Supplementary Tables S1 and S2). All of them were located in the ligand-binding ectodomain, but only three lied close to the predicted functional sites (<10 Å). Five of these non-conservative substitutions were consistent with the above-mentioned radical substitutions changing the physicochemical properties of the molecules. This was different than in hTLRs, where out of the six non-conservative substitutions detected by PROVEAN (one in hTLR3, one in hTLR4 and five in hTLR5), only three were located in the ectodomain, while the other three lied in the signalling TIR domain. Two of these substitutions were STOP codons (one in hTLR4 and one in hTLR5), three radically changed the residuum properties and only one was conservative (Supplementary Table S2).

### Detection of positive selection acting on *TLRs*

In total, we identified five sites in chTLR4 (A26V, R261K, K343R, Y383H and Q611R) and two in chTLR7 (I121V and S447G) that were under significant positive selection (IFEL: p < 0.05; FUBAR: posterior probability > 0.95; Supplementary Table S3). Two sites in chTLR4 (Y383H and Q611R) and the two sites in chTLR7 were recognised based on the two independent statistical approaches. In addition, we found three sites in chTLR3 (S17F, S399R, K513R), one site in chTLR4 (S23C) and two sites in chTLR5 (S409T and A619E) where positive selection was detected with only marginal non-significance (IFEL: 0.05 < p < 0.1; FUBAR: posterior probability 0.90 < p < 0.95). In contrast, we identified only two putatively positively selected sites in hTLRs (hTLR4: T399I; hTLR5: I644F), both being marginally non-significant in the tests. All positively selected sites in both species were located in the ligand-binding ectodomains, with the exception of I644F in hTLR5 which lay in the transmembrane domain (Fig. 3). None of the positively selected sites was consistent with the non-conservative sites with putatively dramatic functional effects detected by the PROVEAN analysis (Supplementary Table S2 and S3).

### Allelic variability in *TLRs* and its distribution across chicken breeds

We identified 1.2× to 6.4× higher levels of *TLR* haplotype diversity (Hd) in chickens than in humans (Fig. 1b, Table 1; for a complete list of *chTLR* alleles and corresponding protein variants see Supplementary Table S4). Only in *hTLR7* (site 665), *chTLR3* (sites 495 and 1781), *chTLR4* (site 903) and *chTLR5* (site 2001) was there significant evidence for recombination between alleles (p < 0.01; confirmed by both SBP and GARD methods). Species-specific difference in allele number had a clear impact on the shape of the haplotype networks, with networks constructed from *hTLR* sequences exhibiting star-like patterns and networks constructed from *chTLR* sequences showing far more complex patterns (Supplementary Fig. S2). Haplotype diversity ranged from 0.244 to 0.759 in *hTLRs*, being in all cases lower than in *chTLRs* (Hd range 0.849–0.947; Table 1). The variation of individual human populations is generally low over all the receptors (Fig. S2). Similar to the nucleotide diversity, also in the case of haplotype diversity our results do not appear to be affected by the particular selection of the 110 individuals in the chicken or human population. In chickens, the effect of variation in the numbers of individuals across the breeds (the same six breeds as above) on the haplotype diversity was minor: *chTLR3* Hd_110_ = 0.903, Hd_(2 ind × 6 br)_ = 0.877, Hd_(4 ind × 6 br)_ = 0.892, Hd_(6 ind × 6 br)_ = 0.885; *chTLR4* Hd_110_ = 0.947, Hd_(2 ind × 6 br)_ = 0.913, Hd_(4 ind × 6 br)_ = 0.941, Hd_(6 ind × 6 br)_ = 0.942; *chTLR5* Hd_110_ = 0.854, Hd_(2 ind × 6 br)_ = 0.855, Hd_(4 ind × 6 br)_ = 0.864, Hd_(6 ind × 6 br)_ = 0.863; *chTLR7* Hd_110_ = 0.849, Hd_(2 ind × 6 br)_ = 0.855, Hd_(4 ind × 6 br)_ = 0.825, Hd_(6 ind × 6 br)_ = 0.824, and increasing the human dataset to the full sample of 2504 people represented in the 1000 Genomes Project did not importantly alter our estimate of the human *TLR* haplotype diversity: *hTLR3* Hd_110_ = 0.759 vs. Hd_2504_ = 0.747, *hTLR4* Hd_110_ = 0.244 vs. Hd_2504_ = 0.289, *hTLR5* Hd_110_ = 0.709 vs. Hd_2504_ = 0.711, and *hTLR7* Hd_110_ = 0.481 vs. Hd_2504_ = 0.481.

In *chTLRs*, many alleles were shared between chicken breeds (Supplementary Fig. S2 and S3 and Supplementary Table S4). Despite this allele sharing, distinguishable population structure was revealed based on both neutral loci (19 microsatellites) and *TLRs* alleles (Supplementary Fig. S4). Unlike microsatellites, however, variation in the allelic frequency of *chTLRs* did not allow detailed resolution of breed identity with increasing number of dividing groups (K).

### Convergent evolution in *chTLRs*

**Figure 4:**
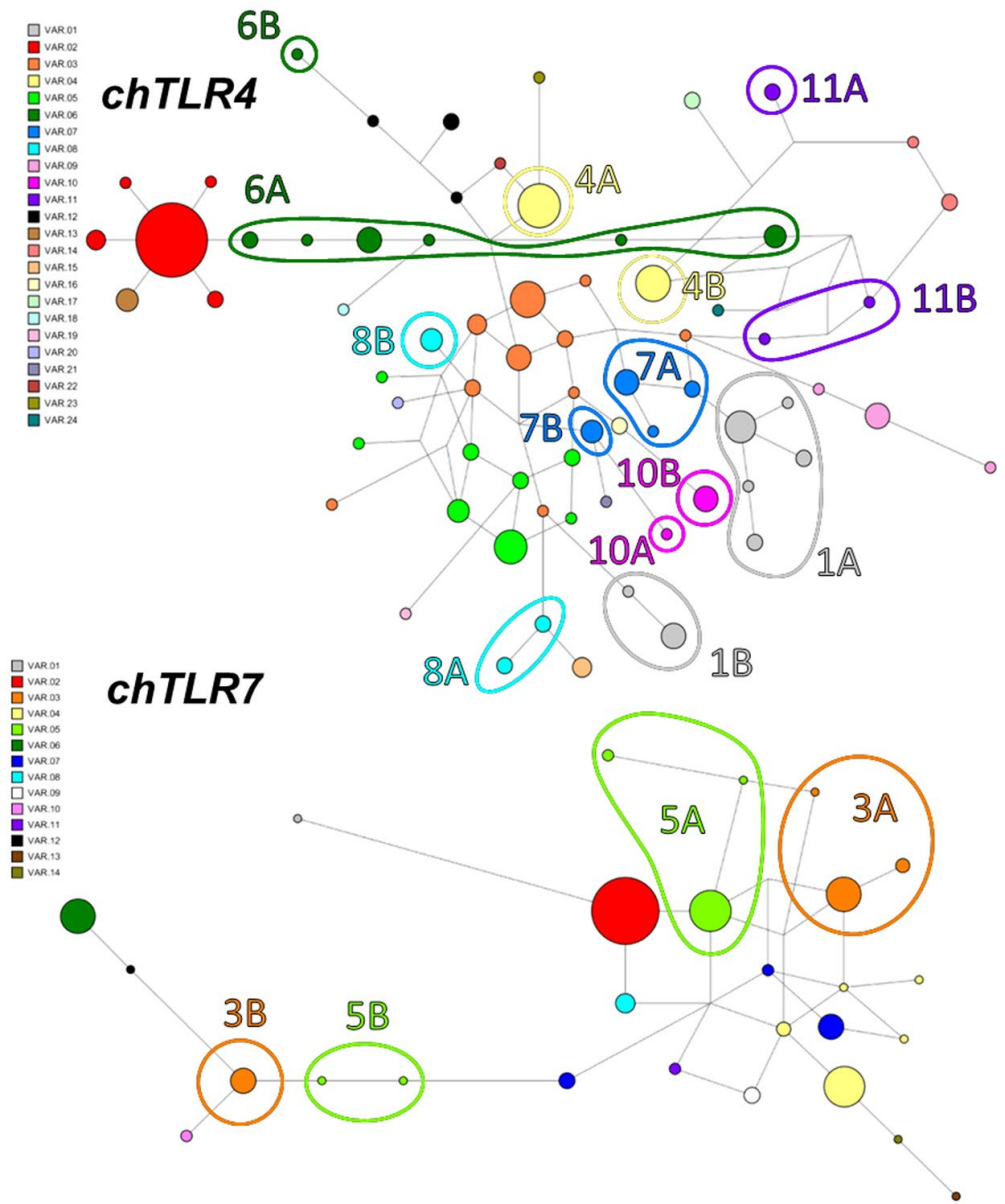
Convergent evolution in chicken *TLR4* and *TLR7*, as indicated by identical protein variants originating from unrelated alleles. Alleles encoding the same protein variant are marked in the haplotype network with the same colour. Distinct clusters of convergent alleles are numbered, highlighted with circles and marked with A or B. (For distances between clusters see Supplementary Table S5).

We detected signals of convergent evolution in *chTLR* alleles, giving origin to six protein variants in chTLR4 and two in chTLR7 (Fig. 4, Supplementary Table S5). In contrast, no signs of convergence were identified in human *TLRs* or any other chicken *TLRs*.

## Discussion

Immune-related genes involved in host defence are remarkable for their high levels of genetic polymorphism. Although typically investigated in genes of the vertebrate Major Histocompatibility Complex *(MHC)*, innate immune receptors may also harbour striking levels of interspecific as well as intraspecific evolutionarily adaptive variation. Our results indicate that while this pattern remains elusive in human innate immune genes it can be clearly documented in domestic fowl. Since their domestication from wild red junglefowl *(Gallus gallus)* at least 4000 years ago^44,45^, chickens have evolved in a shared environment with humans^46^, coming into contact with joint microbial challenges including viral (e.g. avian influenza viruses^47^) or bacterial infections (e.g. *Salmonella*^48^, *Campylobacter*^49^; *Helicobacter*^50,51^ and others^52^). However, probably due to differences in their ancestral populations and distinct modes of recent selection, the two species are differentially equipped to face these threats. Therefore, chickens and humans may respond very differently to the same set of pathogens that infect both species (e.g. when infected with gastrointestinal pathogens^53^). Here we show that chicken *TLR (chTLR)* genes from population sets of comparable sizes exhibit up to 20× higher nucleotide diversity in up to 6× more alleles than human *TLRs (hTLRs)*. As an example, Georgel *et al*. detected only four *TLR4* alleles in a French human population^54^, compared with the 70 *TLR4* alleles now detected in European chickens.

Previous genomic research has shown that, in general, the chicken genome is about 2× richer in exon polymorphism than the human genome^13,14^. Interestingly, we found even higher levels of genetic variation in chicken *TLRs*, with up to 4× more single nucleotide variants (SNVs) in *chTLRs* than *hTLRs*. The levels of human *TLR* nucleotide diversity indicated in our study (0.00012–0.00044) are fully comparable to those previously reported by Barreiro *et al*. (0.00027–0.00071)^55^. While the nucleotide diversity we report for *chTLRs* (0.00106–0.00302) is about 30× lower than that in the variable region of the human (0.024–0.071)^56^, chicken (0.0371–0.0400)^57^ and red junglefowl *MHC* (0.068–0.101)^58^, it is still far higher than either the gene average^59^ or levels typical for non-coding regions in humans^60^. Thus, though the distinct patterns of *TLR* variation observed in humans and chickens partially reflect the general features of their genomes, the contrast is higher than expected. This is further supported by the contrasting trend in mitochondrial *HVS-I* variation, which is almost 4× richer in human SNVs. Interestingly, it was in this same *HVS-I* region that the lower genetic variation of humans compared to other primates was first documented^61^; yet here we observe higher variation in humans than chickens. Our results show that while inter-breed differences (not tested in this study) change with increasing sample size within the breeds, the above-mentioned difference between species described in our dataset holds true even with changing sample sizes used for the analysis. This importantly supports the plausibility of our conclusions. However, we do not claim that the difference we found between human and chicken *TLRs* represents a general difference between birds and mammals. In wild house mouse *TLR4* the levels of nucleotide diversity were previously found intermediate to the values detected in this study for humans and chickens^62^.

*TLR5*, with 22 human SNVs and 19 chicken SNVs, appears to break the general trend outlined above. In order to fully comprehend *TLR5* variability, however, it is important to consider SNV frequency. Since 86% of *hTLR5* variability comprises very rare variants, final nucleotide diversity (π) in *TLR5* is actually more than 2× higher in chickens than humans. This pattern suggests weaker negative selection in *hTLR5* compared to other *hTLRs*. In agreement with this hypothesis, STOP-codon-encoding substitutions have been identified in *hTLR5*^63^ and some avian species^64^. We found no *chTLR5* pseudogenes, suggesting a conservative essential function in chickens.

Only around 60% of chicken protein-coding genes are known to possess a single human orthologue^65^. There are several *TLRs* with orthologues missing or with several paralogues^66^. Therefore, to provide a biologically meaningful human-chicken comparison, the *TLR* genes investigated in this study were selected to represent direct orthologues with confirmed and conserved ligand-specificity. In chickens, most nsSNVs were located in regions encoding the ectodomains, suggesting a potential significance for TLR ligand-binding specificity. We recorded 26 non-synonymous (ns) SNVs that have an important impact on residue physicochemical properties, and 13 amino acid substitutions topologically neighbouring receptor functional sites. Of these, five amino acid substitutions (V68D, S89F, Q92K and A646S in chTLR3 and G413S in chTLR5) belong to both these groups and represent candidate sites putatively altering ligand specificity. Furthermore, the S89F variation in TLR3 was identified as a non-conservative substitution by the PROVEAN analysis.

The functional significance of some nsSNVs in chickens is further supported by the results of selection analysis. In contrast to hTLRs, we identified significant positive selection acting on the ligand-binding ectodomains in chTLR4 and chTLR7. While the functional significance of the putative positively selected TLR7 positions remains unclear, there are several candidate sites in TLR4 with high support for a functional effect. In particular, sites 261 and 343 are topological neighbours to the predicted MD-2-dimmerisation and LPS-binding residues^67,68^. Site 343 has previously been identified as under positive selection at the interspecific level in Galloanserae^40^, with positive selection at neighbouring sites also being reported in other avian and mammalian taxa^39,69,70^. Positions 383 and 611 in chTLR4, which harbour non-conservative substitutions that have been recognised as positively selected using several independent approaches (despite lying out of the predicted functional sites), also appear to be relevant. Sites adjacent to position 611 have also been reported as under selection in other vertebrates^39,69^. Finally, residues 383 and 611 have been identified as responsible for differences in salmonellosis resistance in chickens^42^.

Ongoing positive selection, therefore, appears stronger in chickens than humans, as supported by the distinct SNV frequency and allelic variability patterns in *chTLRs* and *hTLRs*. Since both SNVs and alleles show highly skewed frequency distributions in *hTLRs*, while those in *chTLRs* exhibit more equal SNV and allele representation, we assume that negative selection on *TLRs* is stronger in humans, while *chTLRs* appear to be under stronger balancing selection. From this perspective, *chTLRs* are closer in their co-evolution with pathogens to *MHC* than *hTLRs*^71^. Additional support for this view can be gained from the star-like *hTLR* haplotype network patterns (typical for post-selective-sweep populations). In contrast, the complex *chTLR* net-like patterns are indicative of either recombination or convergence. Correspondingly, haplotype diversity is low in *hTLRs*, while the levels in *chTLRs* resemble those of *MHC*^57^. Despite the generally higher recombination rates reported for the chicken genome^65^, our evidence of only four recombination breakpoints in 154 *TLR* alleles does not suggest that high levels of *chTLR* allelic variability were gained through recombination. Hence, convergent evolution may have been more important in *chTLR* evolution, especially in *TLR4*.

Of the total number of 154 *chTLR* alleles in four genes, 100 did not occur in more than one breed. Most of these 100 were low frequency alleles. In contrast, alleles that are more frequent are largely shared between breeds, which may have resulted from ancestral polymorphism maintained across selective breeding events or from inter-breed gene flow. Nevertheless, population structure is distinguishable, with both neutral microsatellites and *TLR*s suggesting differentiation in the breeds. This confirms previous reports of differentiation in chicken breeds using microsatellites^8^, though the shared polymorphism in *chTLRs* seems to be higher than in neutral markers. Apparently, some of the investigated chicken breeds possess high genetic diversity that could not be reliably fully captured with the limited sample used in this study. Therefore, this study has no ambition to compare the breeds one to another. Yet, recognising this phenomenon of inter-breed variation in immunogenetic variability, our study opens the way to further targeted research in larger population datasets.

The emergence of the domestic chicken was associated with inter(sub)specific hybridisation^44,72,73^ increasing their genetic diversity, similarly to humans^74^. Through their recent evolutionary history, both humans and chickens have been selected for survival under similar novel pathogenic environments. Unlike humans, that have been shown to exhibit relatively low levels of genetic variation^61^, chickens may be more diversified given their heterogeneous origin and artificial selection for diversity based on human cultural and economic needs. Taken together, our study documents that the broad spectrum of geographically distinct, locally-adapted chicken populations maintains tremendous immunogenetic variation in *TLRs*. Shaped by natural selection, the diversity shown by chicken *TLRs* far exceeds that in human *TLRs*, providing also an insight into the genetic basis of breed-specific variation in resistance to various infectious diseases^75^. While the data from chickens and humans cannot be directly generalised to birds and mammals, our results highlight that there are important interspecific differences in the levels of putatively functional genetic polymorphism in the innate immune receptors across vertebrates.

## Methods

### Genetic samples

Genetic samples of 110 chickens representing 25 randomly chosen traditional domestic breeds were provided by hobby breeders from the Czech Republic, Slovakia, Germany, France and Italy. The breeds selected were reported to have their origin in the Indian subcontinent, southeast Asia, Europe and South America^76,77^. Although the relatedness of these breeds is unknown and the pedigree information is missing, the breed-flock variation secured sampling of unrelated individuals. For each breed, 1–14 tissue (blood, feather or muscle) samples (average n = 4.4) were collected and stored in 96% ethanol at −20 °C. After analysis, all samples were deposited in the Genetic Bank of the Department of Zoology, Charles University, Prague (GRbio Institution Code: ZCU). For detailed information on the samples used see Supplementary Dataset 1. This research was carried out in accordance with Czech legislation (Act No 246/1992 Coll., on the protection of animals against cruelty) and approved by the Ethical Committee of the Faculty of Science, Charles University (Reference no. 34712/2010–30).

### Molecular analysis of sequence variation in selected chicken genes

For sequencing of complete protein-coding DNA sequences (CDSs) of four chicken *TLRs* (*chTLRs*; 2691 bp of *chTLR3*, 2532 bp of *chTLR4*, 2586 bp of *chTLR5* and 3180 bp of *chTLR7*) and 1–521 bp of hypervariable segment I (*HVS-I*; reference GenBank ID NC_001323.1) of the mitochondrial DNA control region describing the main chicken mitochondrial haplogroups^44^ we used Sanger sequencing that provides longer reads than most NGS techniques. Alleles were identified using PHASE^78^ and verified by cloning. Unique *chTLR* allele sequences were deposited in GenBank (IDs listed in Supplementary Table S4). For detailed protocols see Supplementary Methods online.

### Sequence variation in selected human genes

Variability of human *TLRs* (*hTLRs*) and *HVS-I* was assessed based on sequence data available through the 1000 Genomes Project Phase 3^43^, downloaded using the Data Slicer Tool. We extracted all *hTLR* variable sites in CDSs for all individuals. Due to the necessity of including two alleles of *hTLR7*, which is localised on the X chromosome in humans, only females were pre-filtered. A subset of 110 individuals from 25 populations was then randomly selected in R v. 3.2.1^79^. As with chickens, the population sample size varied around an average of n = 4.4 individuals per population. For detailed information on the human sequences used see Supplementary Methods online.

### Genetic diversity measurements in sequence data

Nucleotide diversity (π), average number of nucleotide differences (k) and haplotype diversity (Hd) for all selected *TLRs* and *HVS-I* were calculated in DnaSP v. 5.10.01^78^. Haplotype networks for all *TLRs* were constructed in Network v. 4.6.1.2. (Fluxus Technology) using a median-joining algorithm^80^. Additional properties, such as breed/population and *TLR* protein variant, were visualised in Network publisher v. 2.0.0.1 (Fluxus Technology). Alignments were screened for recombination breakpoints using the SBP (Single Breakpoint) and GARD (Genetic Algorithm for Recombination Detection) tools, available on the Datamonkey server^81^.

### Prediction of the effects of amino acid substitutions on protein function

PROVEAN (Protein Variation Effect Analyzer) v 1.1^82^ was used to predict the functional effect of the amino acid substitutions detected in the TLRs in our data set. The algorithm blasted and used between 181 and 265 homologous sequences from the NCBI NR protein database to predict the level of conservation of the individual sites in the particular TLRs. As a threshold for the effect on biological function we took the default value of delta alignment score of −2.5.

### Selection analysis

Positive selection was tested using tools available on the Datamonkey server^83^. Individual sites under diversifying and purifying selection were detected using the codon-based maximum likelihood methods, FUBAR^84^ and IFEL. Results with P-values < 0.05 (IFEL) or posterior probabilities > 95% (FUBAR) were considered significant.

### Protein structures

In order to ascertain the precise location of the variation in proteins, we adopted the approach previously described by Vinkler *et al*.^40^. Briefly, amino acid sequences coding the ligand-binding ectodomains of *chTLRs* were used to generate 3D models in the I-TASSER on-line tool^85^. Since proteolytic cleavage between leucine rich repeat 14 and 15 in the TLR7 endosomal domain has been shown to be essential for murine activation^86^ and human^87^ TLR7, we modelled the two parts of TLR7 separately. Models with highest C-scores were selected for analysis. Functional sites reported in the literature were visualised together with the variable sites, the distances between them being measured using the PyMOL Molecular Graphics System v. 1.5 (Schrödinger, LLC). The accuracy of the 3D models was checked using the ModFOLD Model Quality Assessment Server v. 4.0^88^. All structures achieved high levels of confidence at P < 0.05 and Global model quality scores > 0.384. Details on amino acid classification adopted are provided in Supplementary Methods online.

### Analysis of chicken population structure

The sample set of 110 chickens were genotyped for 19 unlinked microsatellite markers. Genetic population structure for both *chTLR* alleles and microsatellites was determined based on the Bayesian clustering approach implemented in STRUCTURE v. 2.3.4^89^. For detailed description of our approach see Supplementary Methods online.

## Electronic supplementary material

Supplementary Information

Dataset 1

## Data Availability

Unique *chTLR* allele sequences were deposited in GenBank (GenBank IDs KU235138- KU235484).
