# Supplementary material for: Avian Toll-like receptor allelic diversity far exceeds human polymorphism: an insight from domestic chicken breeds
Source: Sci Rep. 2018 Dec 14;8:17878. doi: 10.1038/s41598-018-36226-1 (PMC6294777; doi:10.1038/s41598-018-36226-1)
Supplement: Supplementary file 1 — Supplementary Information [file 41598_2018_36226_MOESM1_ESM.pdf]

Avian Toll-like receptor allelic diversity far exceeds human polymorphism: an insight from domestic chicken breeds

Authors: Zuzana Świderská, Adéla Šmídová, Lucie Buchtová, Anna Bryjová, Anežka Fabiánová, Pavel Munclinger, Michal Vinkler

Content

Supplementary Figures........2
Supplementary Tables........6
Supplementary Methods........26
Genetic analysis of chicken Toll-like receptors (*chTLRs*) and Hypervariable segment I (HVS-I)........26
Genetic analysis of human Toll-like receptors (*hTLRs*) and HVS-I........27
Classification of amino acid substitutions........27
Microsatellites in chickens........27
Population structure........28
Supplementary Methods: Figures........29
Supplementary Methods: Tables........30
References........33

Supplementary Figures

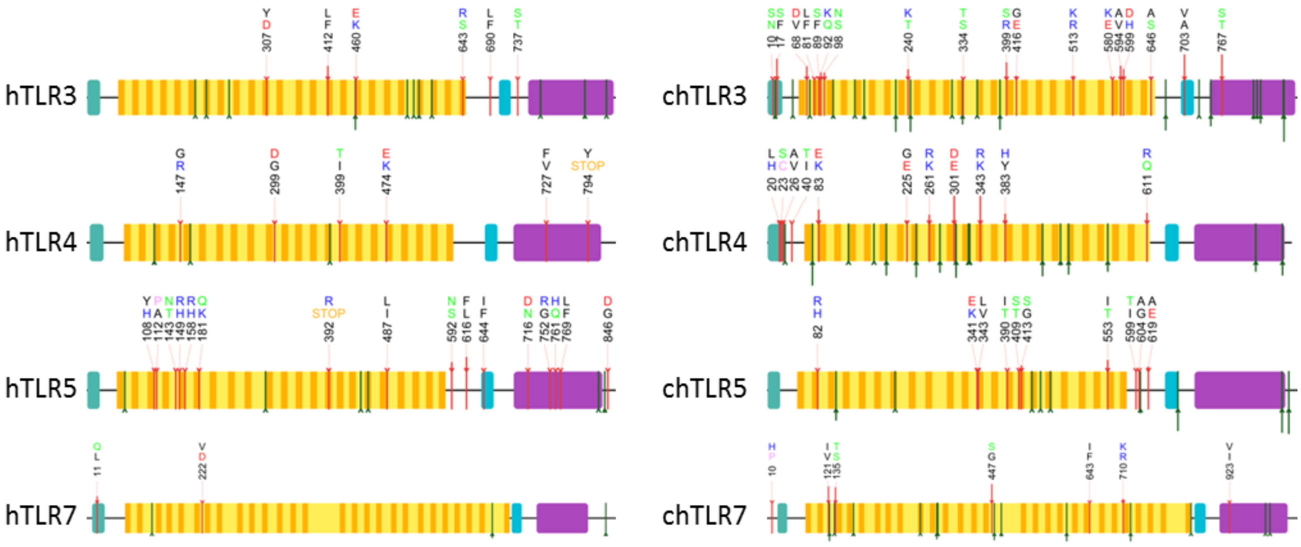

Figure S1. Localisation of single nucleotide variation (SNV) in the linear structure of chicken (ch) and human (h) *Toll-like receptors* (*TLRs*). Regions encoding signal peptides are coloured teal, highly conserved segments of leucine rich repeats (LRRs) orange, variable segments of LRR yellow, transmembrane domains cyan and Toll/interleukin-1 receptor (TIR) domains purple. Non-synonymous SNVs are indicated above with a red arrow and synonymous SNVs below with a dark green arrow. Length of the arrow is proportional to the frequency of minor variants in the SNV. Non-synonymous SNVs are labelled with their position numbers in the amino acid sequence and amino acid variants. Amino acid symbols are coloured according to their physicochemical properties: hydrophobic (A, F, G, I, L, M, V, W, Y) in black, polar uncharged (N, Q, S, T) in green, positively charged (H, K, R) in blue, polar negatively charged (D, E) in red, special cases (C, P) in pink and premature stop codons in orange. The location of signal peptides was predicted using SignalP 4.0[1], LRRs using LRRfinder (http://www.lrrfinder.com), TIR domains using SMART 7 (http://smart.embl-heidelberg.de/);

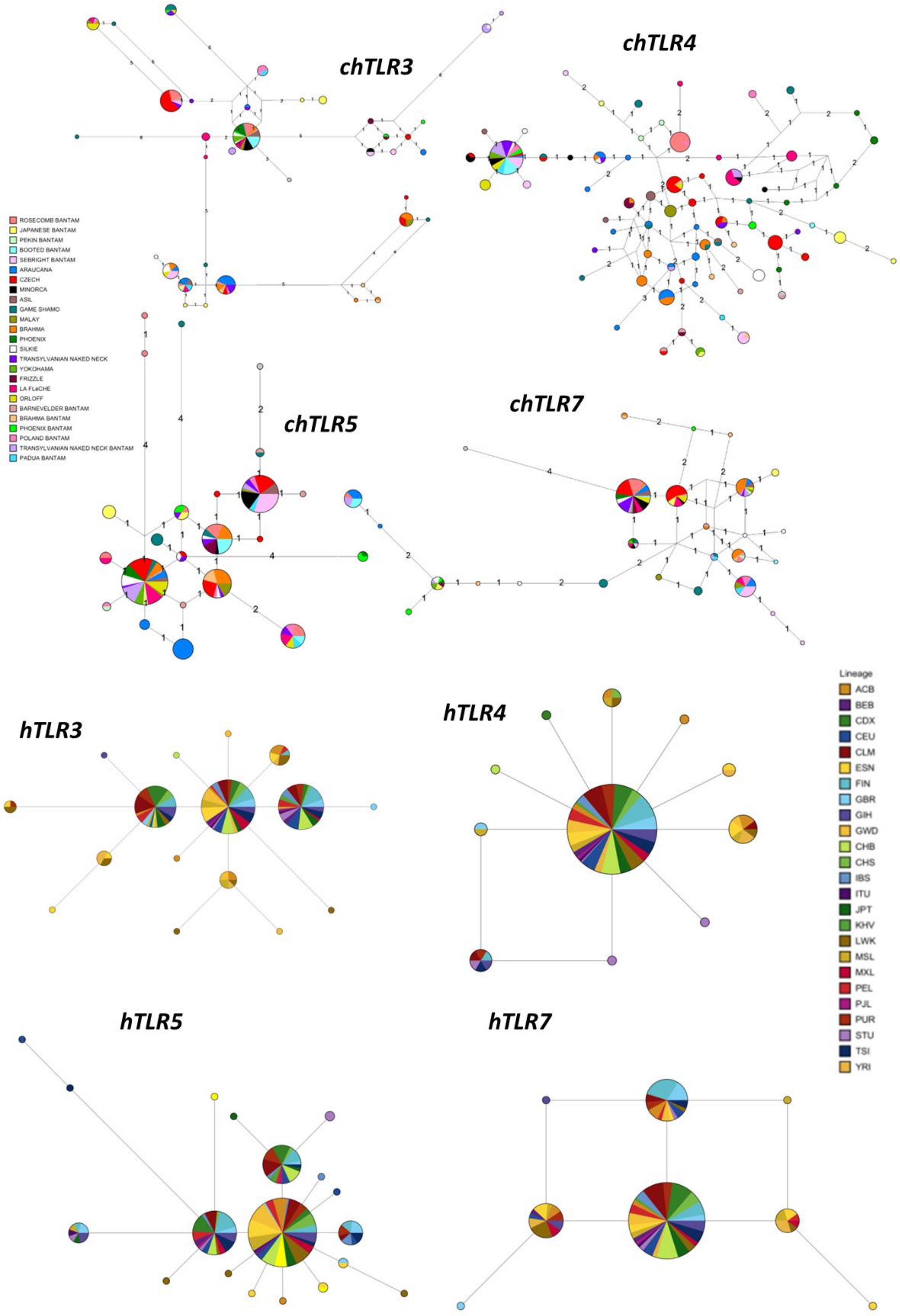

Figure S2. Chicken (ch) and human (h) *Toll-like receptor* (*TLR*) haplotype networks. Pie charts represent unique alleles, with the radius of each pie chart proportional to the frequency of the allele. Lengths of the connecting lines are proportional to the number of pairwise differences between joined haplotypes (also indicated by the number above each line). Pie chart segments are coloured according to the corresponding chicken breed or human population (see in-figure legend on the left or S1 Dataset).

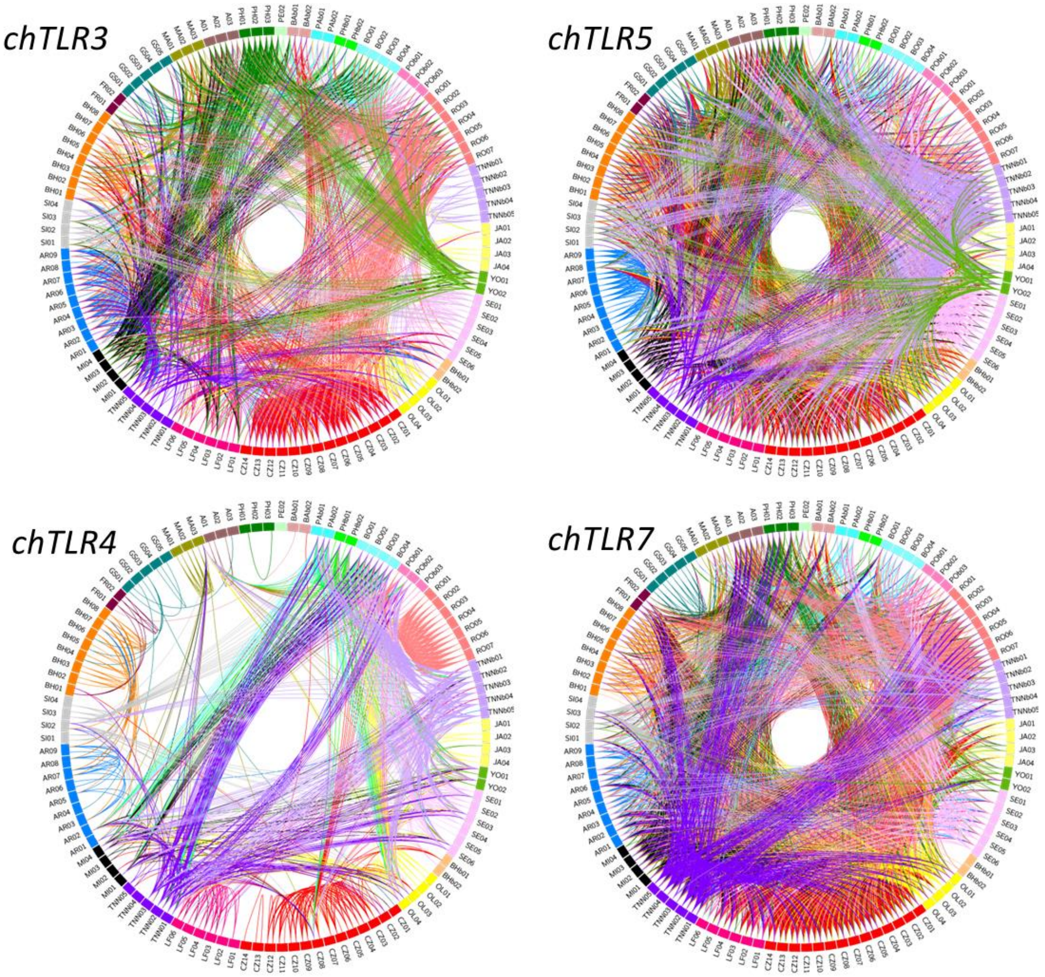

Figure S3. An illustration of *Toll-like receptor* (*TLR*) allele sharing between individuals of different chicken breeds produced using the Circos program[2]. Individuals are grouped based on the breed listed around the circle. Animals sharing the same alleles are interconnected with coloured lines. A = Asil, AR = Araucana, BAb = Barnevelder bantam, BH = Brahma, BHb = Brahma bantam, BO = Booted Bantam, CZ = Czech Golden Pencilled, FR = Frizzle, GS = Game Shamo, JA = Japanese Bantam, LF = La Flèche, MA = Malay, MI = Minorca, OL = Orloff, PAb = Padua bantam, PE = Pekin Bantam, PH = Phoenix, PHb = Phoenix bantam, POb = Poland bantam, RO = Rosecomb Bantam, SE = Sebright, SI = Silkie, TNN = Transylvanian Naked Neck, TNNb = Transylvanian Naked Neck bantam, YO = Yokohama;

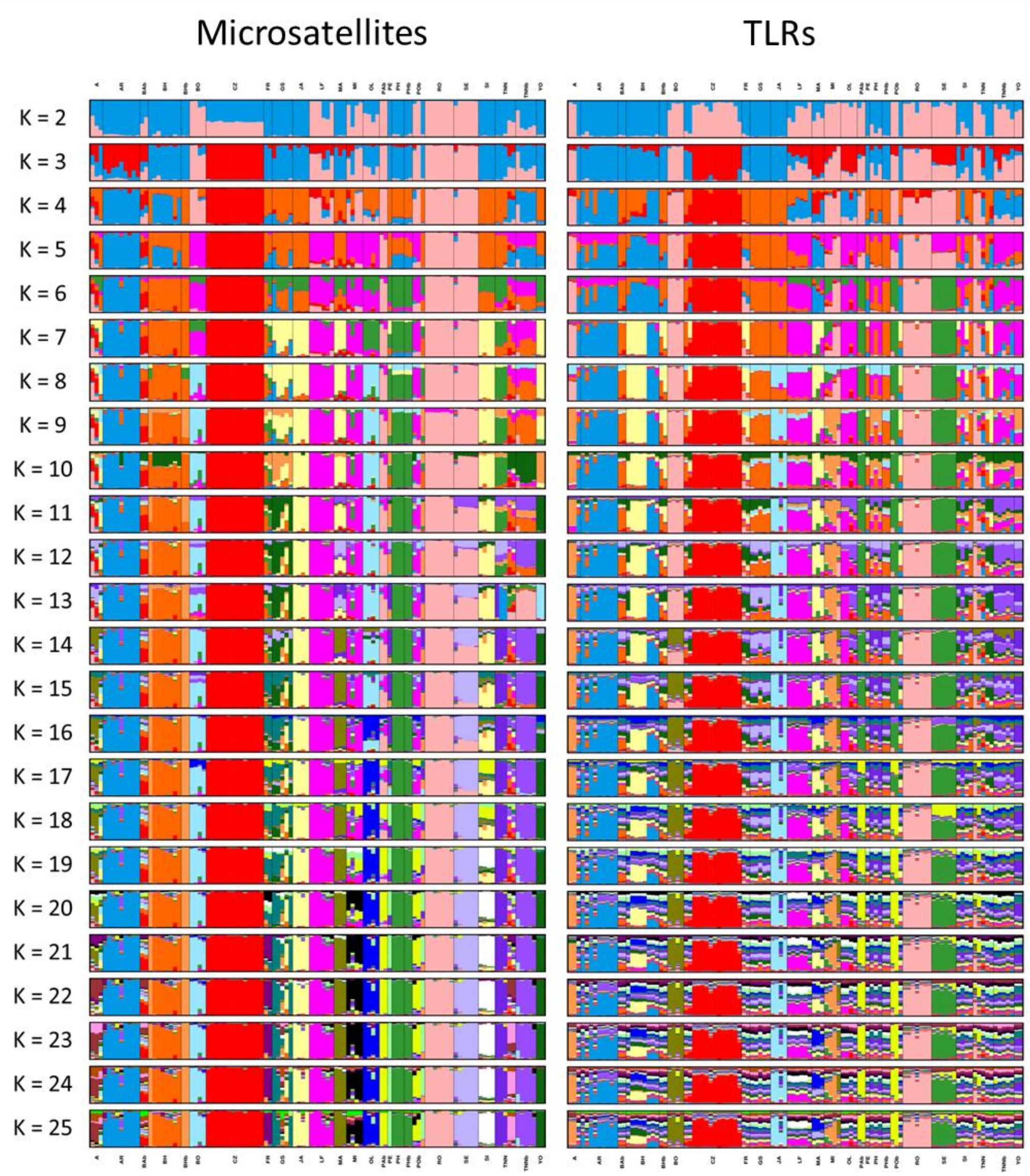

Figure S4. Chicken population structure reconstructed based on Neutral microsatellites and *Toll-like receptor* (*TLR*) alleles. Number of groups to which individuals are divided (K) is indicated on the right, groups are indicated with different colours with their number increasing from the top to bottom. Individuals are ordered according to breed (separated by black lines); breeds are marked with abreviations above and below the diagram: A = Asil, AR = Araucana, BAb = Barnevelder bantam, BH = Brahma, BHb = Brahma bantam, BO = Booted Bantam, CZ = Czech Golden Pencilled, FR = Frizzle, GS = Game Shamo, JA = Japanese Bantam, LF = La Flèche, MA = Malay, MI = Minorca, OL = Orloff, PAb = Padua bantam, PE = Pekin Bantam, PH = Phoenix, PHb = Phoenix bantam, POb = Poland bantam, RO = Rosecomb Bantam, SE = Sebright, SI = Silkie, TNN = Transylvanian Naked Neck, TNNb = Transylvanian Naked Neck bantam, YO = Yokohama.

Supplementary Tables

Table S1. Genetic variation observed in chicken *Toll-like receptors* (*chTLRs*). Genetic variants were determined according to their ancestral state (for details see SI Material and Methods). Single nucleotide variation sites with minor frequency > 1 % (i.e. single nucleotide polymorphisms; SNP) are highlighted in bold. Type of amino acid replacement is marked as radical (Rad.) or conservative (Con.) according to the physicochemical properties of alternative amino acid side chains. Substitutions were classified by PROVEAN analysis to be putatively either "neutral" (Neutr.) or "deleterious" (Delet.) according to their delta alignment score (Table S2). In our study, "deleterious" in the PROVEAN analysis is interpreted as non-conservative and may mean change of the function (e.g. change in the molecule binding in the ligand-binding region of the receptor), but not necessarily functional impairment. Minimum distance means that the minimum distance of a substituted amino acid to any amino acid belonging to the predicted functional site is less than indicated. We used the following sequences as references (GenBank IDs): *chTLR3* - NM_001011691.3, *chTLR4* - NM_001030693.1, *chTLR5* - NM_001024586.1, and *chTLR7* - NM_001011688.2). *Gallus* variants were described by Downing et al.[3]. AA = amino acid, SNV = single nucleotide variant, TM = transmembrane domain, LRR = leucine rich repeat, VS = variable sequence, HCS = highly conserved sequence, ref. = reference, RJF = red junglefowl (*Gallus gallus*), GnJF = green junglefowl (*Gallus varius*), SJF = Sri Lankan junglefowl (*Gallus lafayettii*), GyJF = grey junglefowl (*Gallus sonneratii*), A = Asil, AR = Araucana, BAb = Barnevelder bantam, BH = Brahma, BHb = Brahma bantam, BO = Booted Bantam, CZ = Czech Golden Pencilled, FR = Frizzle, GS = Game Shamo, JA = Japanese Bantam, LF = La Flèche, MA = Malay, MI = Minorca, OL = Orloff, PAb = Padua bantam, PE = Pekin Bantam, PH = Phoenix, PHb = Phoenix bantam, POb = Poland bantam, RO = Rosecomb Bantam, SE = Sebright, SI = Silkie, TNN = Transylvanian Naked Neck, TNNb = Transylvanian Naked Neck bantam, YO = Yokohama.

Gene	SNV position	Sequence change	AA replacement	Domain	Type of AA replacement	PROVEAN prediction	Minimum distance(Å)	Frequency (%)	Alleles with derived variant	Breeds containing derived nt variant	Other studies	dbSNP	Gallus variability			
													RJF	GnJf	SJF	GyJF
TLR3	29	AGC→AAC	S10N	signal	Con.	Neut.	–	0.5	37	TNNb		novel	G	G	G	G
TLR3	**40**	A→C	silent	–	–	–	–	**5.9**	16, 26, 37	PAb, POb, SI, TNNb		rs80605562	A	A	A	A
TLR3	**50**	TCT→TTT	S17F	signal	Rad.	Neut.	–	**39.1**	1-5, 8, 21, 22, 29, 31, 32, 35	A, AR, BAb, BH, BO, FR, GS, LF, MA, MI, OL, PE, PH, PHb, RO, SE, SI, TNN, TNNb, YO		rs80694638	C/T	T	C	C
TLR3	**132**	A→T	silent	–	–	–	–	**2.7**	26, 37	SI, TNNb		rs80753723	A	A	A	A
TLR3	**203**	GTT→GAT	V68D	LRR1-VS	Rad.	Neut.	8	**75.0**	1-6, 8-12, 16-18, 20-26, 29, 31, 35-37	A, AR, BAb, BH, BO, CZ, FR, GS, JA, LF, MA, MI, OL, PAb, PE, PH, PHb, POb, RO, SE, SI, TNN, TNNb, YO	(4)	rs80692549	T/A	A	T	T/A
TLR3	219	C→T	silent	–	–	–	–	0.5	32	GS		novel	C	C	C	C
TLR3	**243**	TTA→TTC	L81F	LRR2-HCS	Con.	Delet.	10	**1.4**	29	TNNb		novel	A	A	A	A
TLR3	266	TCT→TTT	S89F	LRR2-VS	Rad.	Delet.	4	0.5	35	SI		novel	C	C	C	C
TLR3	**274**	CAA→AAA	Q92K	LRR2-VS	Rad.	Neut.	10	**86.4**	1-12, 16-18, 20-26, 29-31, 35-37	in all breeds	(4)	rs14479722	C/A	A	C	C
TLR3	**293**	AAT→AGT	N98S	LRR2-VS	Con.	Neut.	17	**13.2**	13-15, 19, 27, 28, 33, 34	BH, BHb, CZ, GS, LF, MA, OL, TNNb		rs14479723	A/G	A	A	A
TLR3	**339**	T→C	silent	–	–	–	–	**95.9**	1-19, 21-23, 25-37	in all breeds	(4)	rs80589777	C	C	C	C
TLR3	447	G→A	silent	–	–	–	–	0.5	32	GS		novel	G	G	G	G
TLR3	**495**	A→T	silent	–	–	–	–	**6.8**	14, 15, 19, 27, 33	BH, BHb, GS, LF, MA, OL, TNNb		rs80695473	A	A	A	A
TLR3	**657**	A→G	silent	–	–	–	–	**30.5**	4, 5, 7-12, 14, 27, 28, 32, 35	A, AR, BAb, BH, BHb, CZ, GS, JA, LF, OL, PAb, PE, POb, SE, SI, TNN	(4)	rs80630123	A/G	A	A/G	A
TLR3	**719**	AAA→ACA	K240T	LRR8-VS	Rad.	Neut.	33	**19.5**	6, 19, 34	BAb, BH, CZ, LF, MA, OL, RO, SE,		rs80619479	A/C	A	A	A

Gene	SNV position	Sequence change	AA replacement	Domain	Type of AA replacement	PROVEAN prediction	Minimum distance(Å)	Frequency (%)	Alleles with derived variant	Breeds containing derived nt variant	Other studies	dbSNP	Gallus variability			
													RJF	GnJf	SJF	GyJF
										TNN, TNNb						
TLR3	**732**	A→G	silent	–	–	–	–	**30.5**	4, 5, 7-12, 14, 27, 28, 32, 35	A, AR, BAb, BH, BHb, CZ, GS, JA, LF, OL, PAb, PE, POb, SE, SI, TNN	(4)	rs80733702	A/G	A	A/G	A/G
TLR3	**945**	A→G	silent	–	–	–	–	**97.3**	1-25, 27-36	in all breeds	(4)	novel	G	G	G	G
TLR3	**999**	T→G	silent	–	–	–	–	**16.4**	3, 13, 15-18, 20, 34	AR, BH, CZ, GS, JA, MA, OL, PAb, PHb, POb, TNN, TNNb		rs80738024	T/G	G	T	T
TLR3	**1000**	ACT→TCT	T334S	LRR12-HCS	Con.	Neut.	35	**3.6**	20	GS, OL, PHb, TNN		rs80572088	A	T	A	A
TLR3	**1047**	G→A	silent	–	–	–	–	**2.7**	26, 37	SI, TNNb		novel	G	G	G	G
TLR3	**1164**	T→C	silent	–	–	–	–	**26.4**	5, 7-12, 35	A, AR, BAb, BH, BHb, CZ, GS, JA, LF, OL, PAb, PE, POb, SE, SI, TNN	(4)	rs80749871	T/C	T	T	T
TLR3	**1197**	AGC→AGG	S399R	LRR14-VS	Rad.	Neut.	33	**32.3**	7-12, 21-25, 30, 32, 35	A, AR, BAb, BH, BHb, CZ, FR, GS, JA, LF, MI, OL, PAb, PE, PHb, POb, SE, SI, TNN	(4)	rs80713467	C/G	C	C/G	C
TLR3	**1247**	GGG→GAG	G416E	LRR15-VS	Rad.	Delet.	28	**6.8**	21-25, 30, 31	AR, CZ, FR, MI, PHb, SE		rs80718952	G/A	G	G/A	G/A
TLR3	1326	G→A	silent	–	–	–	–	0.0	Only in ref. NM001011691.3			novel	G	G	G	G
TLR3	1329	T→C	silent	–	–	–	–	0.0	Only in ref. NM001011691.3			novel	T	T	T	T
TLR3	**1538**	AAG→AGG	K513R	LRR19-VS	Con.	Neut.	12	**7.3**	21-25, 30-32	AR, CZ, FR, GS, MI, PHb, SE	(4)	rs314125162	A/G	A	A/G	A
TLR3	**1738**	AAA→GAA	K580E	LRR22-HCS	Rad.	Neut.	18	**1.4**	27, 28	BH, BHb		novel	A	A	A	A
TLR3	**1781**	GCG→GTG	A594V	LRR22-VS	Con.	Neut.	8	**25.9**	7-12, 35	A, AR, BAb, BH, BHb, CZ, GS, JA, LF, OL, PAb, PE, POb, SE, SI, TNN	(4)	rs80778047	C/T	C	C	C
TLR3	**1795**	GAC→CAC	D599H	LRR22-VS	Rad.	Delet.	17	**20.0**	6, 19, 33, 36	BAb, BH, CZ, GS, LF, MA, OL, RO,		rs80668052	G/C	G	G	G

Gene	SNV position	Sequence change	AA replacement	Domain	Type of AA replacement	PROVEAN prediction	Minimum distance(Å)	Frequency (%)	Alleles with derived variant	Breeds containing derived nt variant	Other studies	dbSNP	Gallus variability			
													RJF	GnJF	SJF	GyJF
										SE, TNN, TNNb						
TLR3	**1936**	GCT→TCT	A646S	LRR24-VS	Rad.	Neut.	6	**5.0**	19, 33	BH, GS, LF, MA, OL, TNNb		rs80636705	G/T	G	G	G
TLR3	**2013**	A→G	silent	–	–	–	–	**72.3**	1, 2, 4, 5, 7-15, 17, 18, 21-32, 34, 35, 37	in all breeds	(4)	rs80682282	A/G	A	G	G
TLR3	**2108**	GTT→GCT	V703A	TM	Con.	Neut.	–	**47.3**	7-15, 20-28, 30, 31, 34, 35, 37	A, AR, BAb, BH, BHb, CZ, FR, GS, JA, LF, MA, MI, OL, PAb, PE, PHb, POb, SE, SI, TNN, TNNb	(4)	rs80641674	T/C	C	T/C	T
TLR3	**2184**	T→C	silent	–	–	–	–	**2.3**	17, 18	JA		novel	T	T	T	T
TLR3	**2244**	T→C	silent	–	–	–	–	**80.0**	1-5, 7-18, 20-32, 34, 35, 37	in all breeds	(4)	rs316147248	T/C	C	C	C
TLR3	**2299**	TCT→ACT	S767T	TIR	Con.	Neut.	–	**47.3**	7-15, 20-28, 30, 31, 34, 35, 37	A, AR, BAb, BH, BHb, CZ, FR, GS, JA, LF, MA, MI, OL, PAb, PE, PHb, POb, SE, SI, TNN, TNNb	(4)	rs80747649	T/A	A	T/A	T/A
TLR3	**2463**	G→A	silent	–	–	–	–	**6.8**	21, 23, 24, 26, 30, 31, 37	AR, CZ, FR, PHb, SI, TNNb		novel	G/A	G	G	G
TLR3	**2481**	T→A	silent	–	–	–	–	**6.4**	13, 15, 34	BH, CZ, GS, MA		rs80726550	T	T	T	T
TLR3	**2499**	A→G	silent	–	–	–	–	**85.5**	1-7, 10, 12-34, 36, 37	in all breeds	(4)	rs80725644	A/G	G	G	G
TLR3	**2616**	A→G	silent	–	–	–	–	**2.7**	11, 12, 18	JA		novel	A	A	A	A
TLR3	**2619**	C→T	silent	–	–	–	–	**50.9**	1-6, 10, 16, 19, 29, 32, 33, 36	A, AR, BAb, BH, BO, CZ, FR, GS, LF, MA, MI, OL, PAb, PE, PH, PHb, POb, RO, SE, SI, TNN, TNNb, YO	(4)	rs80676425	C/T	C	C	C
TLR4	**59**	CTC→CAC	L20H	signal	Rad.	Neut.	–	**1.8**	58	OL		novel	T	T	T	T
TLR4	**68**	TCC→TGC	S23C	signal	Rad.	Neut.	–	**3.2**	55-57, 63, 68	GS, JA, PE, POb		novel	C	C	C	C

Gene	SNV position	Sequence change	AA replacement	Domain	Type of AA replacement	PROVEAN prediction	Minimum distance(Å)	Frequency (%)	Alleles with derived variant	Breeds containing derived nt variant	Other studies	dbSNP	Gallus variability			
													RJF	GnJf	SJF	GyJF
TLR4	**77**	GCA→GTA	A26V	signal	Con.	Neut.	–	**2.7**	61, 64-66	AR, JA, YO		novel	C	C	C	C
TLR4	**87**	C→T	silent	–	–	–	–	**1.4**	39, 64, 65	AR, SE		novel	C	C	C	C
TLR4	**119**	ACA→ATA	T40I	non-LRR	Rad.	Neut.	11	**1.4**	59, 60	PH		novel	C	C	C	C
TLR4	**219**	G→A	silent	–	–	–	–	**57.3**	3-6, 8-12, 15, 16, 18-20, 26-29, 35, 37, 40, 42, 43, 45, 48, 51, 53, 54, 58-60, 62, 65, 70	A, AR, BAb, BH, BHb, BO, CZ, GS, JA, LF, MA, MI, OL, PAb, PH, PHb, POb, SE, SI, TNN, TNNb, YO	(5)	rs14102162	A/G	G	G	G
TLR4	**247**	GAA→AAA	E83K	LRR1-VS	Rad.	Neut.	12	**25.0**	8-12, 58, 70	A, AR, BO, CZ, MA, MI, OL, PAb, PHb, POb, SE, SI, TNN, TNNb, YO	(5)	rs14102161	G/A	G	G	G
TLR4	**393**	G→A	silent	–	–	–	–	**2.7**	47, 48	JA		novel	G	G	G	G
TLR4	**516**	T→C	silent	–	–	–	–	**2.7**	42, 52, 59, 60	BHb, GS, PH		novel	T	T	T	T
TLR4	**576**	A→G	silent	–	–	–	–	**24.5**	8, 9, 11, 12, 35, 38, 58	A, AR, BO, CZ, GS, MA, MI, OL, PAb, PHb, POb, SE, SI, TNN, TNNb, YO	(5)	rs313485338	A/G	G	A	G/A
TLR4	**674**	GGA→GAA	G225E	LRR7-VS	Rad.	Delet.	12	**5.5**	44-46, 61, 67	BAb, BH, BHb, CZ, FR, JA, YO		novel	G/A	G	G	G
TLR4	**720**	T→A	silent	–	–	–	–	**27.7**	8-10, 12, 33, 35, 38, 58	A, AR, BH, BO, CZ, GS, MA, MI, OL, PAb, PHb, POb, SE, SI, TNN, TNNb, YO	(5)	rs318223020	T/A	A	T	A/T
TLR4	**782**	AGA→AAA	R261K	LRR9-HCS	Con.	Neut.	6	**15.9**	23, 24, 47-49, 68-70	BO, JA, LF, MI, PE, POb, RO, TNNb	(5)	rs312339277	G	G	G	G
TLR4	**834**	C→T	silent	–	–	–	–	**19.5**	6, 13, 14, 16, 17, 20, 25, 26, 28, 30-32, 46, 65, 66	A, AR, BH, BHb, CZ, FR, GS, OL, TNN, TNNb	(5)	novel	C	C	C	C
TLR4	**903**	GAG→GAT	E301D	LRR10-VS	Con.	Neut.	10	**50.5**	8-12, 23, 24, 33-39, 50, 51, 55-58, 62, 64, 65, 68-70	A, AR, BAb, BH, BO, CZ, GS, JA, LF, MA, MI, OL, PAb, PE, PHb, POb, RO,	(5)	rs15032443	G/T	T	T	T

Gene	SNV position	Sequence change	AA replacement	Domain	Type of AA replacement	PROVEAN prediction	Minimum distance(Å)	Frequency (%)	Alleles with derived variant	Breeds containing derived nt variant	Other studies	dbSNP	Gallus variability			
													RJF	GnJf	SJF	GyJF
										SE, SI, TNN, TNNb, YO						
TLR4	**912**	A→G	silent	–	–	–	–	**71.4**	1-8, 13-32, 36, 37, 39-57, 59-63, 65-70	in all breeds	(5)	rs15032442	G/A	A	G	G/A
TLR4	972	T→C	silent	–	–	–	–	0.5	50	BAb		novel	T	T	T	T
TLR4	**978**	G→A	silent	–	–	–	–	**2.3**	51	SI		novel	G/A	G	G	G
TLR4	**1028**	AAA→AGA	K343R	LRR12-HCS	Con.	Neut.	4	**50.5**	1-7, 13-22, 25-32, 40-51, 61-63, 65-67	A, AR, BAb, BH, BHb, BO, CZ, FR, GS, JA, MA, OL, PAb, PH, PHb, POb, SE, SI, TNN, TNNb, YO	(5)	rs15032441	G/A	A	A	A
TLR4	**1147**	TAC→CAC	Y383H	LRR13-VS	Rad.	Neut.	20	**82.3**	8-39, 44-49, 52-66, 68, 70	in all breeds	(5)	rs15032440	T/C	C	T/C	C/T
TLR4	**1327**	T→C	silent	–	–	–	–	**74.1**	1, 3-6, 8-13, 16, 18-20, 22-25, 32-40, 42, 43, 47-49, 52-60, 63, 64, 68-70	A, AR, BAb, BH, BHb, BO, CZ, GS, JA, LF, MA, MI, OL, PAb, PE, PH, PHb, POb, RO, SE, SI, TNN, TNNb, YO	(5)	rs317177658	C/T	C	T	C
TLR4	**1416**	C→T	silent	–	–	–	–	**6.8**	2, 7, 17, 21, 44, 45, 61	BAb, BHb, CZ, FR, GS, JA, PAb, SE, YO		novel	C/T	C	C	C
TLR4	**1455**	C→G	silent	–	–	–	–	**22.7**	1, 3-6, 18, 24, 32, 36, 37, 43, 47-49, 52-54, 59, 60, 63, 69, 70	BAb, BO, CZ, GS, JA, LF, MI, PH, PHb, POb, TNN, TNNb	(5)	rs15032439	C/G	C	C	C
TLR4	**1644**	A→G	silent	–	–	–	–	**17.7**	2, 7, 21, 24, 37, 44, 45, 52, 54, 55, 59-61, 63, 70	BAb, BHb, CZ, FR, GS, JA, LF, MI, PAb, PH, POb, SE, TNNb, YO	(5)	rs314545479	A/G	A	A	A
TLR4	**1832**	CAG→CGG	Q611R	LRRCT-VS	Rad.	Neut.	22	**80.9**	8-24, 33-64, 66-70	in all breeds	(5)	rs15032438	G/A	G	G	G
TLR4	**2358**	G→A	silent	–	–	–	–	**17.7**	2, 4, 17, 24, 25, 36, 37, 39, 44, 45, 54, 56, 59-61, 63	A, BAb, BHb, CZ, FR, GS, JA, LF, MI, PH, POb, SE,	(5)	rs314648336	G/A	G	G	G

Gene	SNV position	Sequence change	AA replacement	Domain	Type of AA replacement	PROVEAN prediction	Minimum distance(Å)	Frequency (%)	Alleles with derived variant	Breeds containing derived nt variant	Other studies	dbSNP	Gallus variability			
													RJF	GnJf	SJF	GyJF
										TNNb, YO						
TLR4	**2487**	G→T	silent	–	–	–	–	**24.5**	3, 4, 6, 12, 17, 18, 20	AR, BH, BHb, BO, CZ, GS, JA, MI, PH, PHb, SE, TNN		rs315160203	G/T	G	T/G	G
TLR5	**245**	CGC→CAC	R82H	LRR2-HCS	Con.	Neut.	5	**2.3**	17	JA		novel	G	-	G	A
TLR5	**336**	G→C	silent	–	–	–	–	**20.0**	1, 10, 12-14	A, BAb, CZ, GS, MA, MI, PAb, POb, SE, TNN, TNNb	(6)	rs313838337	G/C	-	G	G
TLR5	**624**	C→T	silent	–	–	–	–	**1.4**	19	PH, PHb		novel	C	-	C	C
TLR5	**1021**	AAA→GAA	K341E	LRR12-HCS	Rad.	Neut.	15	**98.6**	1-20	in all breeds		novel	G	-	G	A
TLR5	1027	CTC→GTC	L343V	LRR12-HCS	Con.	Delet.	10	0.9	21, 22	RO		novel	C	-	C	C
TLR5	**1169**	ATA→ACA	I390T	LRR14-HCS	Rad.	Neut.	14	**1.4**	19	PH, PHb		novel	T	-	T	T
TLR5	**1225**	TCT→ACT	S409T	LRR15-HCS	Con.	Neut.	16	**2.3**	19, 21, 22	PH, PHb, RO		novel	T	-	T	T
TLR5	**1237**	GGT→AGT	G413S	LRR15-HCS	Rad.	Neut.	10	**89.5**	1-15, 17, 18, 20	in all breeds		novel	A	-	A	G
TLR5	1249	CTG→ATG	L417M	LRR15-HCS	Con.	Neut.	9	0.0	Only in ref. NM_001024586.1		(6)	novel	C	-	C	C
TLR5	1290	T→A	silent	–	–	–	–	0.5	23	GS		novel	T	-	T	T
TLR5	1332	T→C	silent	–	–	–	–	0.5	23	GS		novel	T	-	T	T
TLR5	**1380**	T→C	silent	–	–	–	–	**1.8**	9	GS		novel	T	-	T	T
TLR5	**1658**	ATA→ACA	I553T	LRR21-HCS	Rad.	Neut.	35	**20.0**	1, 12-15	A, BAb, CZ, GS, MA, MI, PAb, POb, SE, TNN, TNNb	(6)	rs315734824	C	-	T	T
TLR5	1796	ACC→ATC	T599I	LRRCT-VS	Rad.	Delet.	35	0.9	20	PE, POb		novel	C	-	C	C
TLR5	**1811**	GCT→GGT	A604G	LRRCT-VS	Con.	Neut.	29	**1.8**	18	LF, RO		novel	C	-	C	C
TLR5	**1818**	A→G	silent	–	–	–	–	**93.2**	1, 2, 5-23	in all breeds		novel	G	-	G	G
TLR5	**1856**	GCG→GAG	A619E	LRRCT-VS	Rad.	Neut.	46	**8.2**	16, 21	BO, LF, OL, PAb, POb, RO, TNN		rs312365402	C	-	C	C
TLR5	**2001**	G→C	silent	–	–	–	–	**61.4**	1-6, 9, 11, 16, 18-20	A, AR, BAb, BH, BHb, BO, CZ, GS, JA, LF, MA, OL, PAb, PE, PH, PHb, POb, RO, SI, TNN,	(6)	rs315207690	G	-	C	C

Gene	SNV position	Sequence change	AA replacement	Domain	Type of AA replacement	PROVEAN prediction	Minimum distance(Å)	Frequency (%)	Alleles with derived variant	Breeds containing derived nt variant	Other studies	dbSNP	Gallus variability			
													RJF	GnJf	SJF	GyJF
										TNNb, YO						
TLR5	**2508**	C→A	silent	–	–	–	–	**41.8**	2-5, 13, 17, 18, 20-22	A, AR, BAb, BH, CZ, GS, JA, LF, MA, OL, PE, PH, POb, RO, SI, TNN, TNNb, YO	(6)	rs317408912	C	-	A	C
TLR5	**2541**	T→C	silent	–	–	–	–	**44.1**	1, 2, 4, 8, 9, 11, 14, 17-23	A, AR, BAb, BH, CZ, GS, JA, LF, MA, OL, PE, PH, PHb, POb, RO, SI, TNN, TNNb, YO	(6)	rs314753079	C/T	-	C	T
TLR7	29	CAC→CCC	H10P	??	Rad.	Neut.	–	0.9	23	PHb		novel	A	A	A	A
TLR7	272	GCA→GTA	A91V	LRR1-VS	Con.	Neut.	–	0.0	Only in ref. NM001011688.1		(4)	novel	C	C	C	C
TLR7	**361**	ATC→GTC	I121V	LRR2-VS	Con.	Neut.	–	**36.8**	7-11, 17-22, 24, 26, 27	AR, BAb, BH, BHb, BO, GS, LF, MA, MI, PAb, PE, PH, POb, RO, SE, SI, TNNb, YO		rs313827291	G	G	G	G
TLR7	**369**	A→G	silent	–	–	–	–	**16.8**	3, 6, 9-11, 18, 19	A, AR, BAb, BH, BHb, BO, JA, MA, OL, PE, POb, RO, SI, TNN, TNNb		rs315579009	A	A	A	A
TLR7	399	T→A	silent	–	–	–	–	0.5	10	SI		novel	T	T	T	T
TLR7	**403**	ACA→TCA	T135S	LRR2-VS	Con.	Neut.	–	**35.0**	1, 2, 21	A, AR, BAb, BH, BO, CZ, FR, GS, LF, MI, OL, PH, RO, SE, SI, TNN, TNNb, YO	(4)	rs316953874	A	A	A	A
TLR7	**513**	T→A	silent	–	–	–	–	**5.0**	11, 18	BAb, BH, BHb, BO, PE, POb, RO		novel	T	T	T	T
TLR7	**912**	T→C	silent	–	–	–	–	**1.4**	6	JA		novel	T	T	T	T
TLR7	**1011**	T→C	silent	–	–	–	–	**87.3**	1-6, 8-25	in all breeds	(4)	rs14883503	T	T	T	T
TLR7	**1017**	T→C	silent	–	–	–	–	**98.2**	1-3, 5-12, 14, 16-27	in all breeds	(4)	novel	C	C	C	C

Gene	SNV position	Sequence change	AA replacement	Domain	Type of AA replacement	PROVEAN prediction	Minimum distance(Å)	Frequency (%)	Alleles with derived variant	Breeds containing derived nt variant	Other studies	dbSNP	Gallus variability			
													RJF	GnJf	SJF	GyJF
TLR7	**1339**	AGT→GGT	S447G	LRR14-VS	Rad.	Neut.	14	**41.8**	3-11, 17, 22, 23, 25, 27	A, AR, BH, BHb, BO, FR, GS, JA, LF, MA, OL, PAb, PE, PH, PHb, POb, RO, SE, SI, TNN, TNNb, YO		rs14883502	G	G	G	G
TLR7	**1356**	T→C	silent	–	–	–	–	**15.0**	5, 14, 17, 23, 25	AR, BHb, BO, FR, JA, PH, PHb, RO, SI, TNNb, YO		novel	T	T	T	T
TLR7	**1398**	C→T	silent	–	–	–	–	**6.4**	5, 14, 16, 23	BHb, FR, JA, PH, PHb, SI, TNNb, YO		novel	C	C	C	C
TLR7	**1791**	T→C	silent	–	–	–	–	**82.7**	1-4, 6-13, 15, 18, 19, 21, 22, 24, 26, 27	in all breeds	(4)	novel	C	C	C	C
TLR7	1927	ATT→TTT	I643F	LRR20-VS	Con.	Delet.	13	0.9	26, 27	SE		novel	A	A	A	A
TLR7	2005	TCT→CCT	S669P	LRR21-HCS	Rad.	Neut.	12	0.0	Only in ref. NM001011688.1		(4)	novel	T	T	T	T
TLR7	**2129**	AAA→AGA	K710R	LRR22-VS	Con.	Neut.	28	**9.1**	17, 25	AR, BO, RO, TNNb		novel	A	A	A	A
TLR7	**2175**	C→G	silent	–	–	–	–	**17.3**	5, 14, 16, 17, 20, 23, 25	AR, BHb, BO, FR, GS, JA, PH, PHb, RO, SI, TNNb, YO		novel	C	C	C	C
TLR7	2538	C→T	silent	–	–	–	–	0.9	13	BH, BHb		novel	C	C	C	C
TLR7	2629	GTG→ATG	V877M	IC	Con.	Neut.	–	0.0	Only in ref. NM001011688.1		(4)	novel	G	G	G	G
TLR7	**2767**	GTT→ATT	V923I	TIR	Con.	Neut.	–	**2.7**	22, 24	GS, MA		novel	G	G	G	G
TLR7	2772	A→G	silent	–	–	–	–	0.0	Only in ref. NM001011688.1		(4)	novel	A	A	A	A
TLR7	2985	G→A	silent	–	–	–	–	0.9	13	BH, BHb		novel	G	G	G	G
TLR7	**3012**	A→G	silent	–	–	–	–	**1.8**	4, 13, 15	BH, BHb, PHb		novel	A	A	A	A

Table S2. Sites predicted by PROVEAN analysis to have putatively dramatic functional effects on the protein function analysis (PROVEAN delta alignment score lower than the cut-off of −2.5) and their localization in the TLRs. Type of amino acid replacement is marked as "Radical" or "Conservative" according to the physicochemical properties of alternative amino acid side chains. h = human, ch = chicken, ECD = ligand-binding ectodomain, TIR = signalling TIR domain.

Gene	Site	PROVEAN score	Domain	Type of AA replacement
hTLR3	L412F	-3.544	ECD	Conservative
hTLR4	Y794stop	-11.077	TIR	Radical
hTLR5	P112A	-4.774	ECD	Radical
	R392stop	-7.833	ECD	Radical
	D716N	-3.353	TIR	Radical
	R752G	-4.132	TIR	Radical
chTLR3	L81F	-3.402	ECD	Conservative
	S89F	-3.754	ECD	Radical
	G416E	-5.176	ECD	Radical
	D599H	-3.13	ECD	Radical
chTLR4	G225E	-4.751	ECD	Radical
chTLR5	L343V	-2.878	ECD	Conservative
	T599I	-5.433	ECD	Radical
chTLR7	I643F	-2.531	ECD	Conservative

Table S3. Sites identified as under positive selection using internal fixed effects likelihood (IFEL) and fast unconstrained Bayesian approximation (FUBAR) methods. Sites showing significant levels of positive selection ($p < 0.05$ for IFEL or Posterior probability > 0.95 for FUBAR) are marked with an asterisk.

Gene	Site	IFEL	FUBAR
		p-value	Posterior prob.
hTLR4	T399I	-	0.940
hTLR5	I644F	0.088	-
chTLR3	S17F	-	0.908
	S399R	-	0.947
	K513R	-	0.937
chTLR4	S23C	-	0.910
	A26V*	-	0.972
	R261K*	-	0.983
	K343R*	-	0.985
	Y383H*	-	0.978
	Q611R*	0.039	0.989
chTLR5	S409T	-	0.922
	A619E	-	0.927
chTLR7	I121V*	0.028	0.970
	S447G*	0.084	0.968

Table S4. Allelic variability observed in chicken *Toll-like receptor* (*chTLR*) genes. A = Asil, AR = Araucana, BAb = Barnevelder bantam, BH = Brahma, BHb = Brahma bantam, BO = Booted Bantam, CZ = Czech Golden Pencilled, FR = Frizzle, GS = Game Shamo, JA = Japanese Bantam, LF = La Flèche, MA = Malay, MI = Minorca, OL = Orloff, PAb = Padua bantam, PE = Pekin Bantam, PH = Phoenix, PHb = Phoenix bantam, POb = Poland bantam, RO = Rosecomb Bantam, SE = Sebright, SI = Silkie, TNN = Transylvanian Naked Neck, TNNb = Transylvanian Naked Neck bantam, YO = Yokohama

Protein variant	Allele	Frequency (%)	GenBank Acc. No.	Breed
chTLR3-Var.1	chTLR3-Allele1	0.45	NM001011691.3	reference
	chTLR3-Allele2	22.17	KU235138 - KU235152	A, BAb, BH, BO, FR, GS, LF, MA, MI, PE, PH, PHb, RO, SI, YO
	chTLR3-Allele3	0.90	KU235153, KU235154	AR, TNN
	chTLR3-Allele4	1.81	KU235155	LF
	chTLR3-Allele5	0.45	KU235156	LF
chTLR3-Var.2	chTLR3-Allele6	14.48	KU235157 - KU235161	BAb, CZ, RO, SE, TNN
chTLR3-Var.3	chTLR3-Allele7	10.41	KU235162 - KU235168	AR, BAb, BH, BHb, CZ, POb, TNN
chTLR3-Var.4	chTLR3-Allele8	7.69	KU235169 - KU235173	AR, BH, OL, SE, SI
chTLR3-Var.5	chTLR3-Allele9	5.88	KU235174 - KU235181	A, AR, GS, JA, LF, PAb, PE, POb
	chTLR3-Allele10	0.45	KU235182	GS
	chTLR3-Allele11	0.45	KU235183	JA
	chTLR3-Allele12	0.45	KU235184	JA
chTLR3-Var.6	chTLR3-Allele13	5.43	KU235185 - KU235187	BH, CZ, MA
	chTLR3-Allele14	0.45	KU235188	BHb
	chTLR3-Allele15	0.45	KU235189	GS
chTLR3-Var.7	chTLR3-Allele16	3.17	KU235190 - KU235192	PAb, POb, TNNb

Protein variant	Allele	Frequency (%)	GenBank Acc. No.	Breed
	chTLR3-Allele17	0.45	KU235193	JA
	chTLR3-Allele18	1.81	KU235194	JA
chTLR3-Var.8	chTLR3-Allele19	4.52	KU235195 - KU235199	BH, LF, MA, OL, TNNb
chTLR3-Var.9	chTLR3-Allele20	3.62	KU235200 - KU235203	GS, OL, PHb, TNN
chTLR3-Var.10	chTLR3-Allele21	0.90	KU235204, KU235205	FR, PHb
	chTLR3-Allele22	1.81	KU235206, KU235207	MI, SE
chTLR3-Var.11	chTLR3-Allele23	0.90	KU235208	CZ
	chTLR3-Allele24	0.45	KU235209	PHb
	chTLR3-Allele25	0.90	KU235210	SE
chTLR3-Var.12	chTLR3-Allele26	2.26	KU235211, KU235212	SI, TNNb
chTLR3-Var.13	chTLR3-Allele27	0.90	KU235213, KU235214	BH, BHb
	chTLR3-Allele28	0.45	KU235215	BH
chTLR3-Var.14	chTLR3-Allele29	1.36	KU235216	TNNb
chTLR3-Var.15	chTLR3-Allele30	0.90	KU235217	AR
chTLR3-Var.16	chTLR3-Allele31	0.90	KU235218	FR
chTLR3-Var.17	chTLR3-Allele32	0.45	KU235219	GS
chTLR3-Var.18	chTLR3-Allele33	0.45	KU235220	GS
chTLR3-Var.19	chTLR3-Allele34	0.45	KU235221	CZ
chTLR3-Var.20	chTLR3-Allele35	0.45	KU235222	SI
chTLR3-Var.21	chTLR3-Allele36	0.45	KU235223	TNN
chTLR3-Var.22	chTLR3-Allele37	0.45	KU235224	TNNb

Protein variant	Allele	Frequency (%)	GenBank Acc. No.	Breed
chTLR4-Var.1	chTLR4-Allele1	0.90	KU235225, NM_001030693.1	BAb, reference
	chTLR4-Allele2	2.26	KU235226, KU235227	BHb, SE
	chTLR4-Allele3	3.62	KU235228	CZ
	chTLR4-Allele4	0.90	KU235229	CZ
	chTLR4-Allele5	0.45	KU235230	PH
	chTLR4-Allele6	0.45	KU235231	TNN
	chTLR4-Allele7	0.45	KU235232	PAb
chTLR4-Var.2	chTLR4-Allele8	0.45	KU235233	A
	chTLR4-Allele9	19.46	KU235234 - KU235247	AR, BO, CZ, MA, MI, OL, PAb, PHb, POb, SE, SI, TNN, TNNb, YO
	chTLR4-Allele10	1.36	KU235248, KU235249	CZ, MI
	chTLR4-Allele11	0.45	KU235250	SI
	chTLR4-Allele12	0.90	KU235251	SE
chTLR4-Var.3	chTLR4-Allele13	1.36	KU235252	A
	chTLR4-Allele14	0.90	KU235253, KU235254	AR, GS
	chTLR4-Allele15	0.45	KU235255	AR
	chTLR4-Allele16	4.52	KU235256 - KU235258	BHb, CZ, OL
	chTLR4-Allele17	0.45	KU235259	GS
	chTLR4-Allele18	0.45	KU235260	GS
	chTLR4-Allele19	0.90	KU235261	CZ
	chTLR4-Allele20	0.45	KU235262	CZ
	chTLR4-Allele21	0.45	KU235263	CZ

Protein variant	Allele	Frequency (%)	GenBank Acc. No.	Breed
	chTLR4-Allele22	2.26	KU235264	MA
chTLR4-Var.4	chTLR4-Allele23	6.79	KU235265, KU235266	POb, RO
	chTLR4-Allele24	4.52	KU235267 - KU235269	LF, MI, TNNb
chTLR4-Var.5	chTLR4-Allele25	0.45	KU235270	A
	chTLR4-Allele26	4.07	KU235271, KU235272	AR, BH
	chTLR4-Allele27	0.90	KU235273	AR
	chTLR4-Allele28	0.90	KU235274, KU235275	AR, TNNb
	chTLR4-Allele29	0.45	KU235276	AR
	chTLR4-Allele30	0.90	KU235277	BH
	chTLR4-Allele31	1.81	KU235278	BH
	chTLR4-Allele32	0.45	KU235279	TNN
chTLR4-Var.6	chTLR4-Allele33	2.26	KU235280 - KU235283	AR, BH, SI, TNN
	chTLR4-Allele34	0.45	KU235284	AR
	chTLR4-Allele35	0.90	KU235285, KU235286	CZ, GS
	chTLR4-Allele36	0.45	KU235287	LF
	chTLR4-Allele37	1.81	KU235288	LF
	chTLR4-Allele38	0.45	KU235289	MI
	chTLR4-Allele39	0.45	KU235290	SE
chTLR4-Var.7	chTLR4-Allele40	2.26	KU235291 - KU235293	BH, CZ, TNN
	chTLR4-Allele41	1.81	KU235294, KU235295	BH, GS
	chTLR4-Allele42	0.45	KU235296	BHb

Protein variant	Allele	Frequency (%)	GenBank Acc. No.	Breed
	chTLR4-Allele43	0.90	KU235297	PHb
chTLR4-Var.8	chTLR4-Allele44	0.90	KU235298, KU235299	BAb, FR
	chTLR4-Allele45	0.90	KU235300, KU235301	BAb, CZ
	chTLR4-Allele46	1.81	KU235302, KU235303	BH, FR
chTLR4-Var.9	chTLR4-Allele47	2.26	KU235304	JA
	chTLR4-Allele48	0.45	KU235305	JA
	chTLR4-Allele49	0.45	KU235306	BO
chTLR4-Var.10	chTLR4-Allele50	0.45	KU235307	BAb
	chTLR4-Allele51	2.26	KU235308	SI
chTLR4-Var.11	chTLR4-Allele52	0.90	KU235309	GS
	chTLR4-Allele53	0.45	KU235310	PH
	chTLR4-Allele54	0.45	KU235311	PH
chTLR4-Var.12	chTLR4-Allele55	0.90	KU235312	GS
	chTLR4-Allele56	0.45	KU235313	JA
	chTLR4-Allele57	0.45	KU235314	PE
chTLR4-Var.13	chTLR4-Allele58	1.81	KU235315	OL
chTLR4-Var.14	chTLR4-Allele59	0.90	KU235316	PH
	chTLR4-Allele60	0.45	KU235317	PH
chTLR4-Var.15	chTLR4-Allele61	1.36	KU235318, KU235319	JA, YO
chTLR4-Var.16	chTLR4-Allele62	0.90	KU235320, KU235321	A, GS
chTLR4-Var.17	chTLR4-Allele63	0.90	KU235322	POb

Protein variant	Allele	Frequency (%)	GenBank Acc. No.	Breed
chTLR4-Var.18	chTLR4-Allele64	0.45	KU235323	AR
chTLR4-Var.19	chTLR4-Allele65	0.45	KU235324	AR
chTLR4-Var.20	chTLR4-Allele66	0.45	KU235325	AR
chTLR4-Var.21	chTLR4-Allele67	0.45	KU235326	BHb
chTLR4-Var.22	chTLR4-Allele68	0.45	KU235327	PE
chTLR4-Var.23	chTLR4-Allele69	0.45	KU235328	LF
chTLR4-Var.24	chTLR4-Allele70	0.45	KU235329	MI
chTLR5-Var.1	chTLR5-Allele1	0.45	NM_001024586.1	reference
chTLR5-Var.2	chTLR5-Allele2	28.05	KU235330 - KU235345	A, AR, BAb, BH, CZ, GS, JA, LF, MA, OL, PE, PH, SI, TNN, TNNb, YO
	chTLR5-Allele3	5.43	KU235346	AR
	chTLR5-Allele4	1.36	KU235347	AR
	chTLR5-Allele5	0.45	KU235348	BAb
	chTLR5-Allele6	10.86	KU235349 - KU235355	BH, BHb, CZ, MA, POb, SI, TNN
	chTLR5-Allele7	11.76	KU235356 - KU235363	BH, BO, FR, GS, MI, RO, SI, TNN
	chTLR5-Allele8	2.71	KU235364 - KU235367	RO, JA, PHb, TNN
	chTLR5-Allele9	1.81	KU235368	GS
	chTLR5-Allele10	0.45	KU235369	CZ
	chTLR5-Allele11	1.36	KU235370 - KU235372	CZ, SI, TNN
chTLR5-Var.3	chTLR5-Allele12	18.10	KU235373 - KU235381	A, CZ, MA, MI, PAb, POb, SE, TNN, TNNb
	chTLR5-Allele13	0.45	KU235382	BAb
	chTLR5-Allele14	0.90	KU235383, KU235384	BAb, GS

Protein variant	Allele	Frequency (%)	GenBank Acc. No.	Breed
	chTLR5-Allele15	0.45	KU235385	CZ
chTLR5-Var.4	chTLR5-Allele16	7.69	KU235386 - KU235392	BO, LF, OL, PAb, POb, RO, TNN
chTLR5-Var.5	chTLR5-Allele17	2.26	KU235393	JA
chTLR5-Var.6	chTLR5-Allele18	1.81	KU235394, KU235395	LF, RO
chTLR5-Var.7	chTLR5-Allele19	1.36	KU235396, KU235397	PH, PHb
chTLR5-Var.8	chTLR5-Allele20	0.90	KU235398, KU235399	PE, POb
chTLR5-Var.9	chTLR5-Allele21	0.45	KU235400	RO
chTLR5-Var.10	chTLR5-Allele22	0.45	KU235401	RO
chTLR5-Var.11	chTLR5-Allele23	0.45	KU235402	GS
chTLR7-Var.1	chTLR7-Allele1	0.45	NM001011688.1	reference
chTLR7-Var.2	chTLR7-Allele2	32.13	KU235403 - KU235418	A, AR, BAb, BH, CZ, FR, LF, MI, OL, PH, RO, RZ, SI, TNN, TNNb, YO
chTLR7-Var.3	chTLR7-Allele3	8.60	KU235419 - KU235427	A, AR, BH, MA, OL, PE, POb, TNN, TNNb
	chTLR7-Allele4	0.45	KU235428	BHb
	chTLR7-Allele5	4.52	KU235429 - KU235435	FR, JA, PH, PHb, SI, TNNb, YO
	chTLR7-Allele6	1.36	KU235436	JA
chTLR7-Var.4	chTLR7-Allele7	11.76	KU235437 - KU235442	AR, LF, MA, PAb, POb, SE
	chTLR7-Allele8	1.36	KU235443 - KU235445	GS, PAb, TNNb
	chTLR7-Allele9	0.45	KU235446	SI
	chTLR7-Allele10	0.45	KU235447	SI
	chTLR7-Allele11	0.45	KU235448	BO
chTLR7-Var.5	chTLR7-Allele12	12.22	KU235449 - KU235454	BAb, CZ, JA, LF, MI, OL

Protein variant	Allele	Frequency (%)	GenBank Acc. No.	Breed
	chTLR7-Allele13	0.90	KU235455, KU235456	BH, BHb
	chTLR7-Allele14	0.45	KU235457	BHb
	chTLR7-Allele15	0.45	KU235458	PHb
	chTLR7-Allele16	0.45	KU235459	SI
chTLR7-Var.6	chTLR7-Allele17	8.60	KU235460 - KU235463	AR, BO, RO, TNNb
chTLR7-Var.7	chTLR7-Allele18	4.52	KU235464 - KU235469	BAb, BH, BHb, PE, POb, RO
	chTLR7-Allele19	0.90	KU235470, KU235471	BAb, BH
	chTLR7-Allele20	1.81	KU235472	GS
chTLR7-Var.8	chTLR7-Allele21	2.71	KU235473 - KU235478	GS, LF, MI, PH, SE, YO
chTLR7-Var.9	chTLR7-Allele22	1.81	KU235479	GS
chTLR7-Var.10	chTLR7-Allele23	0.90	KU235480	PHb
chTLR7-Var.11	chTLR7-Allele24	0.90	KU235481	MA
chTLR7-Var.12	chTLR7-Allele25	0.45	KU235482	AR
chTLR7-Var.13	chTLR7-Allele26	0.45	KU235483	SE
chTLR7-Var.14	chTLR7-Allele27	0.45	KU235484	SE

Table S5. List of chicken Toll-like receptor (TLR) protein variants with potentially convergent origins.

Gene	Protein variant	Cluster A – Alleles	Cluster B - Alleles	Min. distance A-B	Min. distance of any allele from A cluster to any allele(s) from other PrVar	Min. distance of any allele from B cluster to any allele(s) from other PrVar
TLR4	PrVar01	A1, A3, A4, A5, A6	A2, A7	4 SNVs<br>(A1↔A7)	1 SNVs<br>(A3↔A43)	2 SNVs<br>(A7↔A21)
	PrVar04	A23	A24	3 SNVs<br>(A23↔A24)	1 SNVs<br>(A23↔A68)	2 SNVs<br>(A24↔A36, A37)
	PrVar06	A33, A34, A35, A36, A37, A38	A39	3 SNVs<br>(A36↔A39)	2 SNVs<br>(A34↔A23, A57)	2 SNVs<br>(A39↔A56)
	PrVar07	A40, A42, A43	A41	3 SNVs<br>(A40↔A41)	1 SNVs<br>(A43↔A18)	2 SNVs<br>(A41↔A14, A15, A22)
	PrVar08	A44, A45	A46	4 SNVs<br>(A44↔A46)	2 SNVs<br>(A44↔A21)	1 SNVs<br>(A46↔A14)
	PrVar10	A50	A51	3 SNVs<br>(A50↔A51)	2 SNVs<br>(A50↔A41)	2 SNVs<br>(A51↔A62)
	PrVar11	A52	A53, A54	4 SNVs<br>(A52↔A53, A54)	3 SNVs<br>(A52↔A60)	2 SNVs<br>(A54↔A37, A59)
TLR7	PrVar03	A3, A4, A6	A5	5 SNVs<br>(A3↔A5)	1 SNVs<br>(A3↔A9, A4↔A15)	1 SNVs<br>(A5↔A14)
	PrVar05	A12, A13, A15	A14, A16	3 SNVs<br>(A12↔A16)	1 SNVs<br>(A12↔A2, A15↔A4)	2 SNVs<br>(A16↔A20)

Supplementary Methods

Genetic analysis of chicken Toll-like receptors (*chTLRs*) and Hypervariable segment I (HVS-I)

All polymerase chain reactions (PCR; total volume 10-25 μL) were carried out using the Multiplex PCR Master Mix kit (Qiagen) in according with the manufacturer's instructions, with modifications for particular primer-pairs (see Supplementary Methods Table M1). Complete coding DNA sequences (CDSs) of *chTLRs* were amplified and/or sequenced using primers designed according to reference sequences retrieved from NCBI GenBank (IDs: *chTLR3* - NM_001011691.3, *chTLR4* - NM_001030693.1, *chTLR5* - NM_001024586.1, *chTLR7* - NM_001011688.2). List of used primers with their sequences is provided in Supplementary Methods Table M2 and their localisation is visualised in Supplementary Methods Fig. M1. HVS-I was amplified and sequenced using the previously published primers L16750 (5'- AGGACTACGGCTTGAAAAGC -3')[7] and H522 (5'- ATGTGCCTGACCGAGGAACCAG -3')[8]. PCR products were enzymatically purified with Exonuclease I (New England Biolabs) and Calf Intestinal Alkaline Phosphatase (New England Biolabs) before commercial Sanger sequencing at GATC Biotech (Germany). All sequences were assembled and manually checked in SeqScape v. 2.5 (Applied Biosystems) and aligned in BioEdit v. 7.2.5 using the ClustalW Multiple alignment tool with default settings. No indels were detected in the alignments. The FaBox online toolbox[9] was used to extract all single nucleotide variants (SNVs) from the alignments. Individual-allelic sequences were resolved using the PHASE algorithm implemented in DnaSP v. 5.10.01[10]. This was possible in part because of the high frequency of homozygotes in our dataset, i.e. 14 alleles of *chTLR3* (37.8 %), 19 alleles of *chTLR4* (27.1 %), 10 alleles of *chTLR5* (43.5 %) and 10 alleles of *chTLR7* (37 %). Presence of these alleles allowed identification of the

pairing alleles in heterozygotes. Sequences of alleles in heterozygotes that could not be reliably resolved based on this approach were verified by cloning using the CloneJet kit (Thermo Scientific) with pGEM-T vector (Promega) and JM109 High Efficiency Competent Cells (Promega). At least two different positive clones were sequenced in each case.

Genetic analysis of human Toll-like receptors (*hTLRs*) and HVS-I

The genome ranges of interest according to the GRCh37.p13 Primary Assembly were 4:186997774-186998214, 4:186999994-187000185, 4:187003474-187005326 and 4:187005799-187006027 for hTLR3, 9:120466751-120466843, 9:120470841-120471007 and 9:120474667-120476926 on for hTLR4, 1:223286373-223283797 for hTLR5, X:12885698-12885800 and X:12903631-12906777 for hTLR7 and 16023-16548 of mitochondrial DNA for partial HVS-I, with length corresponding to the mitochondrial sequence used in chickens.

Classification of amino acid substitutions

Amino acids were grouped into five categories based on the physicochemical properties of their side chains, i.e. amino acids with hydrophobic (A, F, G, I, L, M, V, W, Y), polar uncharged (N, Q, S, T), polar positively charged (H, K, R) and polar negatively charged (D, E) side chains, and special cases (C, P). Substitutions of amino acids within groups were considered as conservative, and those between groups as radical.

Microsatellites in chickens

Nineteen unlinked microsatellite loci (ADL20, ADL112, ADL123, ADL142, ADL191, ADL247, ADL262, ADL306, ADL315, COL1A1, LEI64, LEI120, MCW5, MCW83, MCW178, MCW217, MCW219, MCW230 and ROS302) located on 14 different chromosomes were amplified in three multiplex PCRs using the Type-it Microsatellite PCR Kit (Qiagen). A 6 µl reaction volume was used

under the following cycling conditions: initial denaturation at 95°C for 5 minutes, 30 cycles of 95°C for 30 s, 57°C for 90 s, 72°C for 60 s and a final extension at 60°C for 30 minutes with specific primers, of which one was fluorescently labelled (details in Supplementary Methods Table M3). Subsequently, 1 μl of PCR product was mixed with 9 μl of formamide and 0.3 μl GeneScan 500 LIZ dye size standard (Applied Biosystems). This was denatured by heating at 95 °C for 2 minutes and analysed on an Applied Biosystems 3130xl Genetic Analyser. Fragment sizes were determined using GeneMarker software (SoftGenetics). Approximately 0.72 % of all data were missing.

Population structure

Genetic population structure was examined based on the Bayesian clustering approach implemented in STRUCTURE v. 2.3.4[11] with the admixture model, with separate runs for the alleles of TLRs and Neutral markers. We performed 10 independent runs for each K value (ranging from 1 to 25). Each run included 500 000 iterations following a burn-in period of 100 000 iterations. Delta K was calculated in Structure Harvester[12], as described by Evanno et al.[13]. Independent replications for particular K values were combined in CLUMPP[14] and visualised in Distruct[15].

Supplementary Methods Figures

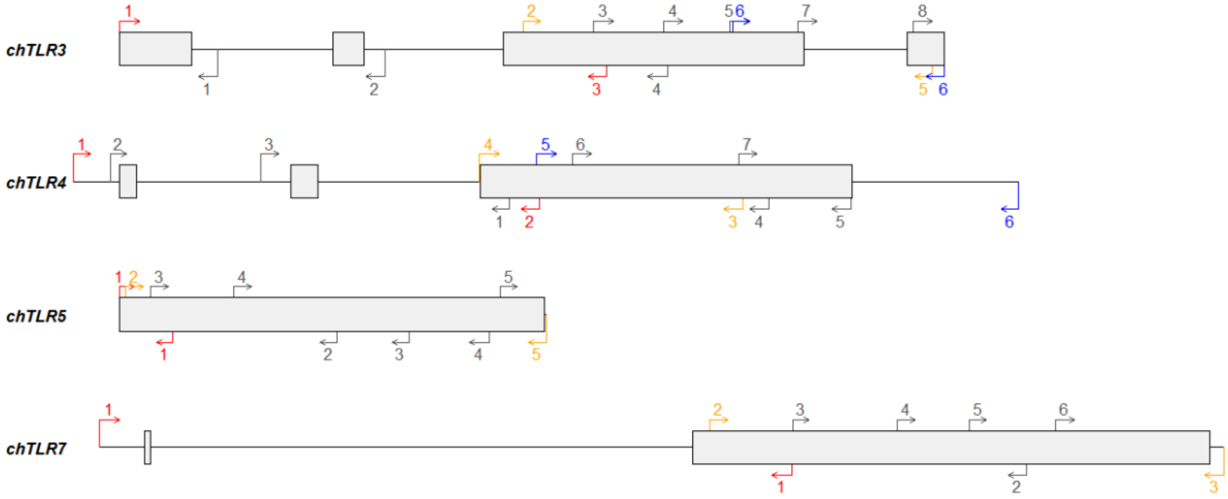

Figure M1. Localisation of primers used in this study. Coding sequences for chicken Toll-like receptors (chTLRs) are displayed as grey boxes; amplification primers are indicated in red, orange or blue (matching primer pairs in the same colour); and primers used for sequencing only in grey. Forward primers lie above the line and reverse primers below. Numbering of the primers corresponds with Supplementary Methods Table M2.

Supplementary Methods Tables

Table M1. Polymerase chain reaction (PCR) conditions used for particular chicken *Toll-like receptor* (*TLR*) PCR products.

Gene	Segment	F primer	R primer	Product length (bp)	Concentration of primers (μM)	Annealing time (s)	Annealing temperature (°C)	Elongation time (s)	Number of cycles
TLR3	1	chTLR3_F1-A1	chTLR3_R3-A1	2959	0.2	40	62	150	35
TLR3	2	chTLR3_F2-A2	chTLR3_R5-A2	2481	0.3	40	61	180	35
TLR3	3	chTLR3_F6-A3	chTLR3_R6-A3	1288	0.3	60	58	150	35
TLR4	1	chTLR4_F1-A1	chTLR4_R2-A1	2830	0.3	30	61	150	35
TLR4	2	chTLR4_F4-A2	chTLR4_R3-A2	1601	0.3	40	60	100	35
TLR4	3	chTLR4_F5-A3	chTLR4_R6-A3	2927	0.2	30	62	150	33
TLR5	1	chTLR5_F1-A1	chTLR5_R1-A1	327	0.3	60	56	30	35
TLR5	2	chTLR5_F2-A2	chTLR5_R5-A2	2554	0.3	60	62	150	35
TLR7	1	chTLR7_F1-A1	chTLR7_R1-A1	4207	0.3	40	62	240	38
TLR7	2	chTLR7_F2-A2	chTLR7_R3-A2	3122	0.3	40	61	150	38
**HVS-I**		L16750	H522	525	0.3	45	60	60	35

Table M2. Primers used for polymerase chain reaction amplification (A) and sequencing (S) of selected chicken *Toll-like receptors* (*TLRs*). Position reported in bp from the start codon.

Gene	Name	Sequence (5' – 3')	Orientation	Position (bp)	Use	Segment
TLR3	chTLR3_F1-A1	ATGGGATGCTCTATTCCTTGCTGGA	Forward	1	A	1
TLR3	chTLR3_R1	CTGACATAATAAGCCACTAGGCC	Reverse	598	S	1
TLR3	chTLR3_R2	GCAGGCTTGATCTTGATAGATGC	Reverse	1616	S	1
TLR3	chTLR3_F2-A2	CTCACTGAGCCATGTGAAGC	Forward	2459	A	2
TLR3	chTLR3_F3	GCTAATTCCAGCTTGCAGGTTCTC	Forward	2886	S	2
TLR3	chTLR3_R3-A1	GGAGGAAAATGCCCCACTTTC	Reverse	2960	A+S	1
TLR3	chTLR3_F4	TTGGAAATGTGCAAACCCAGGAGG	Forward	3308	S	2
TLR3	chTLR3_R4	AGGTCCTCCTGGGTTTGCACATTT	Reverse	3335	S	2
TLR3	chTLR3_F5	CCTAAATATCACGGTACTCTGG	Forward	3714	S	2
TLR3	chTLR3_F6-A3	GTACTCTGGTGTTGCATTTTG	Forward	3727	A	3
TLR3	chTLR3_F7	GTGACTGAACATCTCTTACAGGA	Forward	4125	S	3
TLR3	chTLR3_F8	GTCGAGACTCCATCATACTGATC	Forward	4828	S	3
TLR3	chTLR3_R5-A2	TGGCCAGTTCAAGATGCAGC	Reverse	4940	A+S	2
TLR3	chTLR3_R6-A3	TCAGCGCACTTTACTATTAGTT	Reverse	5015	A+S	3
TLR4	chTLR4_F1-A1	GGCATTCAAGTGACATCACAC	Forward	-276	A	1
TLR4	chTLR4_F2	GTCCTTTGCTCCGCATCCC	Forward	-50	S	1
TLR4	chTLR4_F3	GGTCCTATTAGAAGCAGGACAG	Forward	861	S	1

Gene	Name	Sequence (5' – 3')	Orientation	Position (bp)	Use	Segment
TLR4	chTLR4_F4-A2	GGTGCCACCTCCATACAATAGAAG	Forward	2192	A+S	2
TLR4	chTLR4_R1	GATGGGTAGGTCAGACAGAG	Reverse	2370	S	1
TLR4	chTLR4_F5-A3	AGGCTCAACCTCACGTTGGTACTT	Forward	2539	A+S	3
TLR4	chTLR4_R2-A1	ACGTGAGGTTGAGCCTGTTTGT	Reverse	2554	A	1
TLR4	chTLR4_F6	CTCTTGAGTGGACTGTGTCAGG	Forward	2758	S	2
TLR4	chTLR4_F7	GATATGCCACACACCTGCCTAC	Forward	3768	S	3
TLR4	chTLR4_R3-A2	TCATGTAGGCAGGTGTGTGGCATA	Reverse	3793	A	2
TLR4	chTLR4_R4	CACTGAGCAGCACCAATGAGTAG	Reverse	3949	S	3
TLR4	chTLR4_R5	GAGTTTTATCTCCTCGTGATTCC	Reverse	4446	S	3
TLR4	chTLR4_R6-A3	GCAAGCAAACACAGCATCCAG	Reverse	5466	A+S	3
TLR5	chTLR5_F1-A1	ATGATGTTACATCAACGGC	Forward	1	A	1
TLR5	chTLR5_F2-A2	CACTAGCTGGAGATATATGTGC	Forward	41	A	2
TLR5	chTLR5_F3	GCAACTTCATTTCCACTGCTGG	Forward	190	S	2
TLR5	chTLR5_R1-A1	GAATGTTATTGAACCCCAGG	Reverse	328	A+S	1
TLR5	chTLR5_F4	GGACGTTAGTGAGAATGGCTGG	Forward	699	S	2
TLR5	chTLR5_R2	GGTCACCCAGATTTGCGAGCC	Reverse	1327	S	2
TLR5	chTLR5_R3	GCACAGTCACAGACATACTTG	Reverse	1763	S	2
TLR5	chTLR5_R4	CCAAATGGCATCACGAATATTGTTG	Reverse	2253	S	2
TLR5	chTLR5_F5	GCCTTTAATTTTGCCCAGAGC	Forward	2317	S	2
TLR5	chTLR5_R5-A2	CATCAACTGTCAGTGTGAGAC	Reverse	2595	A+S	2
TLR7	chTLR7_F1-A1	AAAGCAGTGGTTGCTGCTGTTGTC	Forward	-117	A+S	1
TLR7	chTLR7_F2-A2	TTGTGGAGATTGACTTCAGGTG	Forward	3588	A+S	2
TLR7	chTLR7_R1-A1	GCCACTGAGATCAAGAATTTCTAGG	Reverse	4090	A+S	1
TLR7	chTLR7_F3	TGCCCACGTTGCTATAACGC	Forward	4094	S	2
TLR7	chTLR7_F4	GGTCGAAGTTGCAAGTCCAAAG	Forward	4730	S	2
TLR7	chTLR7_F5	GATGGAAAGCCAATCTCTTCA	Forward	5167	S	2
TLR7	chTLR7_R2	CGGAGTATCAGTTCTTGGAGAG	Reverse	5514	S	2
TLR7	chTLR7_F6	GTGATGCAGTATGGTTTGTTGGG	Forward	5688	S	2
TLR7	chTLR7_R3-A2	GCTGCAAATGCTGAATGAGGTG	Reverse	6710	A+S	2

Table M3. Overview of microsatellite markeres used in chickens. Chr: chromosome, MPLX: multiplex membership

Marker	Chr	F primer sequence (5' – 3')	R primer sequence (5' – 3')	MPLX	Fluorophore	Length range (bp)
ADL0020	1	TAGATAAAAATCCTTCCCTT	GCACTCAAAAGAAAACAAAT	2	PET	97 - 112
ADL0112	10	ATCTCAAATGTAATGCGTGC	GGCTTAAGCTGACCCATTAT	1	VIC	123 - 132
ADL0123	11	GCTGTGTCAAGATTAGAATCAC	AACAATGAAAAACACTACCTGA	2	VIC	102 - 141
ADL0142	6	CAGCCAATAGGGATAAAAGC	CTGTAGATGCCAAGGAGTGC	1	6-FAM	227 - 237
ADL0191	9	AAAGGAAAGCCTATGTGAAT	AAAGCACCAAGCGAGATACA	2	6-FAM	133 - 143
ADL0247	5	CTCTTGTTGTCTGTCTTGTG	TGCATGTTGTCAGTTTTCAG	3	VIC	163 - 170
ADL0262	23	GTGCAGACACAGAGGGAAAG	TCACATGCACACAGAGATGC	2	6-FAM	100 - 107
ADL0306	3	GTTACTGTATCTTGGCTCAT	TCAGTTTGACTTTCCTTCAT	3	NED	115 - 133
ADL0315	7	TCCTTGGGCAGTAGTTTCAA	CTCCCATGTTGCTTCTTTAG	3	6-FAM	245 - 249
COL1A1	27	CGGACCATGAATTTGGCATT	TTACTCTCTCCTGTCACGCG	1	NED	112 - 118
LEI0064	7	TGGTTGTCTCAATACAACGTC	CTGTAAAGATTTCTCAGAAACAG	3	PET	285 - 307
LEI0120	15	CGTAACACATGCAACTCAATG	TTAGAATGAAAAGGCTGTTCC	2	VIC	274 - 316
MCW0005	4	ACCTCCTGCTGGCAAATAAATTGC	TCACTTTAGCTCCATCAGGATTCA	1	VIC	210 - 253
MCW0083	3	GCCTTTCACCCATCTTACTGT	TACATTTCAGAAGGAATGTTGC	1	PET	83 - 92
MCW0178	7	ACTGGAATTTTAGGGCAACAG	AACTGTTAGCTAATATGACCTG	1	NED	71 - 93
MCW0217	18	GATCTTTCTGGAACAGATTTC	CTGCACTTGGTTCAGGTTCTG	1	PET	147 - 175
MCW0219	18	CCACAGCTATAAATGCTATAGC	GACATGACTTACTGAAAACTAG	3	NED	222 - 239
MCW0230	11	TGCACAGAGCCAAGCTGCTTC	GATCCTCTGATGGCTGCCG	1	NED	260 - 298
ROS0302	24	CACAGACACCCCCGTACAG	ACACAGCGGTTGGTTATGCC	1	VIC	96 - 111

References

1. Petersen, T. N., Brunak, S., von Heijne, G. & Nielsen, H. SignalP 4.0: discriminating signal peptides from transmembrane regions. *Nat. Methods* **8,** 785–786 (2011).
2. Krzywinski, M. *et al.* Circos: An information aesthetic for comparative genomics. *Genome Res.* **19,** 1639–1645 (2009).
3. Downing, T., Lloyd, A. T., O’Farrelly, C. & Bradley, D. G. The Differential Evolutionary Dynamics of Avian Cytokine and TLR Gene Classes. *J. Immunol.* **184,** 6993–7000 (2010).
4. Ruan, W., An, J. & Wu, Y. Polymorphisms of Chicken TLR3 and 7 in Different Breeds. *PLoS ONE* **10,** e0119967 (2015).
5. Ruan, W. K., Wu, Y. H., An, J. & Zheng, S. J. Polymorphisms of chicken Toll-like receptors 4, 15, and 21 in different breeds. *Poult. Sci.* **91,** 2512–2516 (2012).
6. Ruan, W., Wu, Y. & Zheng, S. J. Different genetic patterns in avian Toll-like receptor (TLR)5 genes. *Mol. Biol. Rep.* **39,** 3419–3426 (2012).
7. Fumihito, A. *et al.* One subspecies of the red junglefowl (Gallus gallus gallus) suffices as the matriarchic ancestor of all domestic breeds. *Proc. Natl. Acad. Sci. U. S. A.* **91,** 12505–12509 (1994).
8. Liu, Y.-P. *et al.* Multiple maternal origins of chickens: out of the Asian jungles. *Mol. Phylogenet. Evol.* **38,** 12–19 (2006).
9. Villesen, P. FaBox: an online toolbox for fasta sequences. *Mol. Ecol. Notes* **7,** 965–968 (2007).
10. Librado, P. & Rozas, J. DnaSP v5: a software for comprehensive analysis of DNA polymorphism data. *Bioinforma. Oxf. Engl.* **25,** 1451–2 (2009).
11. Pritchard, J. K., Stephens, M. & Donnelly, P. Inference of Population Structure Using Multilocus Genotype Data. *Genetics* **155,** 945–959 (2000).

12. Earl, D. A. & vonHoldt, B. M. STRUCTURE HARVESTER: a website and program for visualizing STRUCTURE output and implementing the Evanno method. *Conserv. Genet. Resour.* **4,** 359–361 (2011).

13. Evanno, G., Regnaut, S. & Goudet, J. Detecting the number of clusters of individuals using the software STRUCTURE: a simulation study. *Mol. Ecol.* **14,** 2611–2620 (2005).

14. Jakobsson, M. & Rosenberg, N. A. CLUMPP: a cluster matching and permutation program for dealing with label switching and multimodality in analysis of population structure. *Bioinformatics* **23,** 1801–1806 (2007).

15. Rosenberg, N. A. DISTRUCT: a program for the graphical display of population structure. *Mol. Ecol. Notes* **4,** 137–138 (2004).
